# Rhoptry and Dense Granule Secreted Effectors Regulate CD8^+^ T Cell Recognition of *Toxoplasma gondii* Infected Host Cells

**DOI:** 10.3389/fimmu.2019.02104

**Published:** 2019-09-06

**Authors:** Leah M. Rommereim, Barbara A. Fox, Kiah L. Butler, Viviana Cantillana, Gregory A. Taylor, David J. Bzik

**Affiliations:** ^1^Department of Microbiology and Immunology, Geisel School of Medicine at Dartmouth, Lebanon, NH, United States; ^2^Division of Geriatrics, Departments of Medicine, Molecular Genetics and Microbiology, and Immunology, Center for the Study of Aging and Human Development, Duke University Medical Center, Durham, NC, United States; ^3^Geriatric Research, Education and Clinical Center, VA Medical Center, Durham, NC, United States

**Keywords:** *Toxoplasma gondii*, dense granule, rhoptry, antigen presentation, immunity related GTPases

## Abstract

*Toxoplasma gondii* secretes rhoptry (ROP) and dense granule (GRA) effector proteins to evade host immune clearance mediated by interferon gamma (IFN-γ), immunity-related GTPase (IRG) effectors, and CD8^+^ T cells. Here, we investigated the role of parasite-secreted effectors in regulating host access to parasitophorous vacuole (PV) localized parasite antigens and their presentation to CD8^+^ T cells by the major histocompatibility class I (MHC-I) pathway. Antigen presentation of PV localized parasite antigens by MHC-I was significantly increased in macrophages and/or dendritic cells infected with mutant parasites that lacked expression of secreted GRA (GRA2, GRA3, GRA4, GRA5, GRA7, GRA12) or ROP (ROP5, ROP18) effectors. The ability of various secreted GRA or ROP effectors to suppress antigen presentation by MHC-I was dependent on cell type, expression of IFN-γ, or host IRG effectors. The suppression of antigen presentation by ROP5, ROP18, and GRA7 correlated with a role for these molecules in preventing PV disruption by IFN-γ-activated host IRG effectors. However, GRA2 mediated suppression of antigen presentation was not correlated with PV disruption. In addition, the GRA2 antigen presentation phenotypes were strictly co-dependent on the expression of the GRA6 protein. These results show that MHC-I antigen presentation of PV localized parasite antigens was controlled by mechanisms that were dependent or independent of IRG effector mediated PV disruption. Our findings suggest that the GRA6 protein underpins an important mechanism that enhances CD8^+^ T cell recognition of parasite-infected cells with damaged or ruptured PV membranes. However, in intact PVs, parasite secreted effector proteins that associate with the PV membrane or the intravacuolar network membranes play important roles to actively suppress antigen presentation by MHC-I to reduce CD8^+^ T cell recognition and clearance of *Toxoplasma gondii* infected host cells.

## Introduction

*Toxoplasma gondii* [hereafter, *Toxoplasma*] frequently infects warm-blooded vertebrates including humans (1), yet infection by this parasite typically causes little disease burden due to the development of strong protective CD8^+^ T cell immunity. Recognition of *Toxoplasma*-infected cells by CD8^+^ T cells is critical for immune control of infection by this obligate intracellular pathogen (2). The initiation of CD8^+^ T cell responses during infection is determined by the ability of professional antigen presenting cells to acquire and present antigens in the context of major histocompatibility complex I (MHC-I). *Toxoplasma* infected cells have been observed *in vitro* to present antigen to CD8^+^ T cells (3–5), and perforin mediated cytolysis of parasite infected cells suggests these cells present antigen *in vivo* to prime effector CD8^+^ T cells (6, 7). Surprisingly, professional antigen presenting cells that phagocytosed *Toxoplasma* failed to initiate significant CD8^+^ or CD4^+^ T cell responses *in vitro* or during *in vivo* infection. Active invasion and formation of the parasitophorous vacuole (PV) in infected macrophages and dendritic cells is critical for the priming of significant CD4^+^ and CD8^+^ T cell responses (5).

A unique aspect of *Toxoplasma* biology is parasite replication within a specialized non-fusogenic PV that the parasite forms upon entering a host cell by active invasion (8). Currently identified endogenous CD8^+^ T cell antigens of *Toxoplasma* are dense granule (GRA) or rhoptry (ROP) secreted proteins that reside within the lumen of the PV (9, 10), or that localize to the limiting PV membrane (PVM) (11, 12). Targeting of a model CD8^+^ T cell antigen to different cellular compartments in *Toxoplasma* revealed that antigens localized to the PV lumen were associated with the highest level of presentation by MHC-I in parasite infected host cells (13). Retargeting the ROP5 PVM antigen to the PV lumen resulted in markedly increased antigen presentation of the endogenous CD8^+^ T cell epitope (12). Collectively, these previous studies suggested that *Toxoplasma* antigens associated with the PV initiate the CD8^+^ T cell responses to infection.

*Toxoplasma* antigens are presented via the classical MHC-I pathway once their cognate protein antigen reaches the host cell cytosol (9, 14). Various models have been proposed for antigen release from the PV (15), though the mechanisms that control the release of PV localized antigens to the host cell cytosol for processing and presentation by MHC-I are still unknown. A retro-translocation model is based on the physical association and fusion of the PV membrane with host endoplasmic reticulum (4). Another model suggested the host immunoproteasome could directly access PVM localized parasite proteins (10). Alternatively, parasite antigens may be released from the PV after PVM/PV disruption by host cell autonomous IFN-γ-dependent mechanisms (3, 16, 17). Nonetheless the PVM is unlikely to be an idle target as certain PVM-associated *Toxoplasma* secreted effector molecules participate in parasite mediated mechanisms that effectively resist IFN-γ and immunity related GTPases (IRG) dependent host cell mechanisms that would otherwise effectively degrade the PV to promote parasite clearance (17–26). Understanding the mechanisms that regulate the entry of *Toxoplasma* antigens into the host pathway for presentation by MHC-I and CD8^+^ T cell recognition is important for designing effective vaccines against intracellular pathogens or cancer (27–32).

We hypothesized that host cell mediated disruption of the PV could release PV localized parasite antigens to increase antigen presentation by MHC-I and the recognition of infected cells by CD8^+^ T cells. Consequently, parasite secreted effector proteins which preserve the integrity of the PV may function to suppress antigen presentation by infected host cells. Consistent with this hypothesis, we show that deletion of ROP5 or ROP18 in infected macrophages and dendritic cells, or GRA7 in infected macrophages, increased PV clearance and the presentation of soluble PV antigen by MHC-I molecules. In contrast, deletion of GRA2 increased the presentation of soluble PV antigen by MHC-I molecules, though PV clearance was not affected. Moreover, the increased antigen presentation phenotypes observed in the GRA2 deletion mutant were critically dependent on the expression of the GRA6 protein. Collectively, our results reveal that host cell type, host IFN-γ, and host IRG effectors are targets of multiple rhoptry and dense granule secreted effectors that function in association with PV membranes to dynamically regulate CD8^+^ T cell recognition of *Toxoplasma* infected host cells.

## Materials and Methods

### Ethics Statement

All procedures involving mice were reviewed and approved by the Institutional Animal Care and Use Committee of Dartmouth College (Animal Welfare Assurance Number #A3259-01) and were in accordance with the guidelines published in the Guide for the Care and Use of Laboratory Animals of the National Institutes of Health.

### Mice

C57BL/6 female mice were purchased from Jackson Labs (Bar Harbor ME) and maintained at the Center for Comparative Medicine and Research at the Geisel School of Medicine at Dartmouth in specific-pathogen-free conditions.

### Parasites and Cell Culture

All parasite cultures were maintained *in vitro* by serial passages in human foreskin fibroblast (HFFs) monolayers in Eagle's modified essential medium (EMEM) (Gibco) supplemented with 1% fetal bovine serum (FBS) (Life Technologies) (33).

### Generation of Isogenic OVA-Expressing Knockout and Complemented Parasite Strains

All strains used or developed in this study are listed in Table S1. Knockout targeting constructs were developed and parasites were transfected following the previously described protocol (34). Primers used for construct development and knockout or complementation validation are in Table S2. OVA expressing strains were engineered by amplifying *ptub*P30-OVA by PCR from plasmid *ptub*P30-OVA/*sag*CAT (35) using primers ptub_R and DHFR.3′_F that contain overlaps with the *UPRT* 5′- and 3′- untranslated regions (UTRs) to generate plasmid pOVA, that targets OVA to the UPRT locus following FUDR negative selection (Figure S1A) (34). The Δ*rop5-*OVA and Δ*gra2-*OVA strains were complemented by targeting the WT gene, *rop5C* for Δ*rop5* and *gra2* for Δ*gra2*, to the endogenous gene locus and then using 6TX selection to isolate positive clones (Figures S2A, S2D). The Δ*rop18* strain was complemented with targeting plasmids containing WT ROP18 (*ROP18*), a kinase dead ROP18 (*ROP18*^*KD*^) (36) or a ROP18 protein unable to interact with ATF6β (*ROP18*^*ATF*6β^) (37). Each of the *ROP18* complementation targeting plasmids contained the complementing *ROP18* with a *ROP18* promoter and *ptub*P30-OVA construct flanked by the *UPRT* 5′- and 3′- UTRs to target the plasmid to UPRT in the parental RHΔ*ku80*Δ*rop18::HXGPRT* strain using 5-fluorodeoxyuridine (FUDR) selection to isolate positive clones (Figure S3A). Plasmid pROP18^KD^ was engineered to mutate the aspartic acid to an alanine at position 394 in ROP18 (38) by QuickChange site direct mutagenesis (Agilent). Plasmid pROP18^ATF6β^ was generated with primers that deleted amino acids 147 to 164 in ROP18 (37). Genotype verification of gene replacements and deletion events was assessed by PCR and sequencing as previously described (34).

### Validation of OVA Expressing and Complemented Strains

HFF cells were cultured on coverslips and infected with parasites for 24–48 h. To visualize OVA expression the cultures were fixed with Histochoice (Amresco), and permeabilized with 0.001% Triton X-100 (Sigma). To visualize ROP18 the cultures were fixed with Histochoice and permeabilized in 0.1% saponin for 10 min. To visualize ROP5, cultures were fixed with 4% PFA (Electon Microscopy Sciences) and permeabilized with 0.01% Triton X-100. To visualize GRA2, cultures were fixed with 4% PFA and permeabilized with 0.1% saponin. All samples were blocked with 10% FBS, incubated with primary antibodies (1 h at RT): rabbit α-OVA (1:1,000, Bethyl Laboratories), rabbit α-ROP18 (ROP18-His WA525, 1:500, Sibley laboratory), rabbit α-ROP5 [MO556, 1:3,000, (23)], and mAb α-GRA2 (France-Delauw Laboratory, 1:500). Cultures were washed 3 times with PBS and incubated 1 h at RT with secondary antibodies conjugated to an Alexa Fluor (Invitrogen). Samples were mounted in ProLong Gold with DAPI (Invitrogen) and then imaged with a Nikon A1R SI confocal microscope (Nikon, Inc.). Confocal images were processed with FIJI (39).

### Preparation of Antigen Presenting Cells

Bone marrow derived macrophages (BMMΦ) were differentiated from bone marrow isolated from C57BL/6 WT or Irgm1/m3 knockout (18) mice in a 5-day culture with 30% L929-culture supernatant as previously described (40). Bone marrow derived dendritic cells (BMDC) were differentiated from bone marrow isolated from C57BL/6 or Irgm1/m3 knockout (18) mice in a 9-day culture with GM-CSF (Peprotech) as previously described (41). Purity was verified by FACS analysis (data not shown) and unless otherwise stated, all incubations with BMMΦs or BMDCs were performed at 37°C.

### Flow Cytometry Based Secretion Assay

A previously described flow cytometry based secretion assay was used to measure the accumulation of OVA protein in the lumen of the PV to verify that different mutant strains of *Toxoplasma* expressed and secreted equivalent OVA into the PV lumen (42). BMMΦ and BMDCs were infected with *Toxoplasma* at a MOI of 2.5 and incubated for 24 h. Cells were then washed with PBS, fixed with PFA (4%) and permeabilized with saponin (0.05%) to selectively expose PV lumen proteins for analysis. The expression of OVA was compared to the expression of GRA5 in each PV. To identify the infected cells that were double positive for GRA5 and OVA, processed cells were stained for mouse α-GRA5 (1:1,000, Biotem, TG 17.113) and revealed using AF647 goat anti-mouse IgG (1:500), and stained with rabbit α-OVA (1:1,000, Bethyl) and revealed using AF488 goat anti-rabbit IgG (1:500).

### B3Z T Cell Activation Assay

*In vitro* antigen presentation by BMMΦ and BMDCs was measured using the B3Z CD8^+^ T cell hybridoma that expresses β-galactosidase upon recognition of the SIINFEKL peptide (OVA_257−264_) presented on H-2K^b^-molecules (Provided by N. Shastri, University of California, Berkley) (43). WT and Irgm1/m3 KO BMMΦ or BMDC (10^5^) were seeded on 96-well trays and incubated overnight (BMMΦ) or for 4–6 h (BMDC). To activate cells (primed) prior to infection they were cultured for 4–6 h with IFN-γ (100 U/ml, Peprotech) and TNF-α (10 U/ml, Peprotech). Parasites were added to the APCs at an MOI of 2.5 and incubated for 12 h. Infected cultures were then spun down and washed twice with RMPI medium without phenol red (Gibco) and B3Z cells (10^5^) were added to the culture and incubated for 12 h. Lysis buffer (44) containing CPRG (final concentration 100 μM) was added to the cultures and plates were incubated for 14 h. Absorbance was read at 562 nm, with 650 nm as the reference wavelength. Data is from three experiments, each conducted in triplicate then averaged and normalized with reference to uninfected controls and represented as the fold change compared to an uninfected control or as the percent increase of each knockout compared to RH-OVA for the same condition.

### Parasitophorous Vacuole Clearance Assay

WT and Irgm1/m3^−/−^ BMMΦ and BMDCs (4 × 10^6^) were seeded on 6-well trays overnight or for 6 h, respectively. To quantitate the level of IFN-γ-dependent PV clearance, the *Toxoplasma* infected cells were either incubated for 4–6 h with normal culture media (non-activated cells) or media containing IFN-γ (final concentration of 100 U/ml, Peprotech) and TNF-α (final concentration of 10 U/ml, Peprotech) (primed cells), and then infected with 100 tachyzoites/well and incubated for 6 days at 37°C. The medium was removed from each well and the remaining monolayer was fixed and stained with coomassie brilliant blue to reveal plaques formed from the surviving *Toxoplasma* PVs. The number of plaque forming units (PFUs) per well were counted and the percent killing (PV clearance) was determined by dividing the difference of the number of PFUs in unprimed and primed cells by the total number of PFUs in the unprimed cells. The assay was performed in triplicate in two separate experiments.

### IRG Coating Assay of the PVM

WT and Irgm1/m3^−/−^ APCs were seeded on a coverslip and incubated overnight (BMMΦ) or for 4–6 h (BMDC), then primed with IFN-γ (final concentration of 100 U/ml, Peprotech) and TNF-α (final concentration of 10 U/ml, Peprotech) for 6 h. Coverslips were infected with parasites at an MOI of 4 for 45 min then washed and fixed with 4% PFA (Electron Microscopy Sciences). Cells were then permeabilized with 0.1% saponin (Sigma) and blocked in 10% FBS. For visualization, cultures were incubated with mouse α-GRA5 (1:2,000, Biotem, TG 17.113) and rabbit α-Irgb6 [1:1,000, (18)], then washed and incubated with secondary antibodies anti-mouse Alexa Fluor 568 and anti-rabbit Alexa Fluor 488 (Invitrogen). Coverslips were mounted in ProLong Gold with DAPI (Invitrogen) and imaged at 63x with a Nikon A1R SI confocal microscope (Nikon, Inc.). All images were processed with FIJI (39). A minimum of 500 vacuoles was scored for each strain for quantification of Irgb6 vacuole coating.

### *In vivo* Infections

Groups of mice were infected intraperitoneally with 100,000 (10^5^) or 1,000 (10^3^) tachyzoites in 0.2 ml PBS. Parasite viability was determined by a plaque forming unit (PFU) assay (data not shown).

### Statistics

Statistical analysis was performed using PRISM software (Graphpad Software). Survival was analyzed by the Log-rank (Mantel-Cox) test performed using the Gehan-Breslow-Wilcoxon test; a *P* ≤ 0.05 was considered significant. All other statistical calculations were performed using the non-parametric Mann-Whitney test; a *P* ≤ 0.05 was considered significant.

## Results

### Development of a Δ*ku80* Genetic Model to Evaluate CD8^+^ T Cell Recognition of *Toxoplasma* Infected Host Cells

The model T cell antigen ovalbumin (OVA) was previously engineered as a single copy gene to drive high-level expression and secretion of soluble OVA protein (amino acids 140–386) into the lumen of the parasitophorous vacuole (PV) (3, 13, 35). This OVA transgene was targeted to the *UPRT* locus in the genetically tractable type I strain RHΔ*ku80* background (33, 45) and clones of RH-OVA expressing a single copy of OVA inserted into the *UPRT* locus were isolated after selection in 5-fluorodeoxyuridine (FUDR) (Figures S1A,B) (46). As expected, OVA accumulated specifically in the PV lumen inside of the PV membrane (PVM) and did not accumulate in parasites or in the host cell (Figures S1C,D). This PV localized OVA antigen contains the SIINFEKL epitope that can be presented by MHC-I at the host cell surface to prime H-2k^b^ restricted SIINFEKL specific CD8^+^ T cells if OVA antigen is released from the PV and is correctly processed by the host cell MHC-I antigen presentation machinery.

### Presentation of PV Localized OVA Antigen by MHC-I Is IFN-γ and Irgm1/m3 Dependent in Macrophages and IFN-γ Dependent and Irgm1/m3 Independent in Dendritic Cells

**Figure 1:**
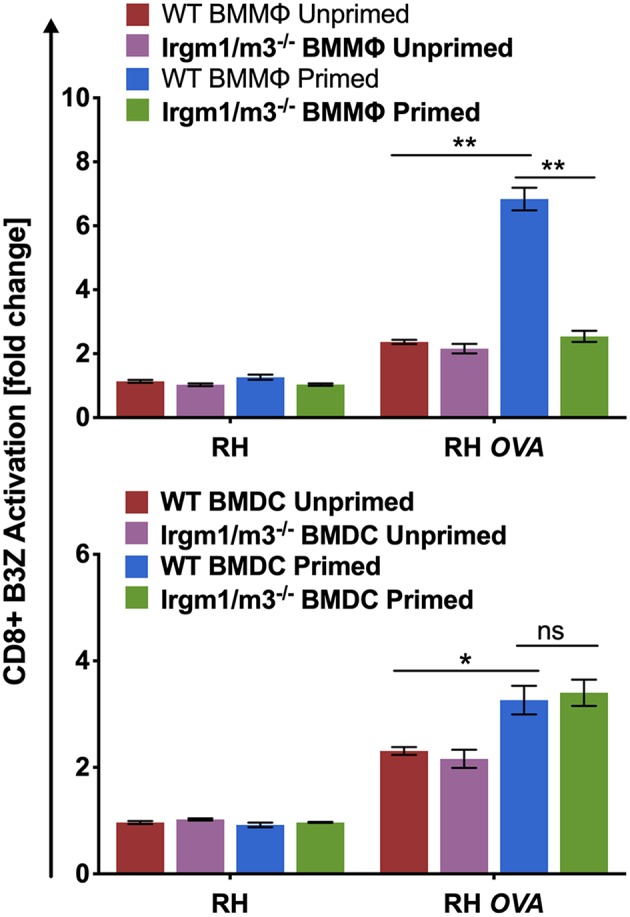
Immunity related GTPases are necessary for optimal antigen presentation in BMMΦs but not in BMDCs. Antigen presentation in WT and Irgm1/m3^−/−^ BMMΦs (*top panel*) or WT and Irgm1/m3^−/−^ BMDCs (*bottom panel*) infected with a wild type non-OVA expressing strain (RH) or OVA-expressing strain (RH-OVA) was determined by CD8^+^ B3Z activation. For statistical analysis activation was compared between specified host cell conditions (WT/Irgm1/m3^−/−^ or primed/unprimed) infected with a specific *Toxoplasma* strain. Mean ± SEM. Mann-Whitney test. *P < 0.05, **P < 0.01.

Irgm regulatory molecules such as Irgm1 and Irgm3 regulate the host cell IRG effector mechanisms that attack the PVM to clear PVs and parasites from infected host cells (16–18, 47). To investigate the role of IFN-γ priming, host cell Irgm1/Irgm3 molecules, and cell type in regulating the presentation of PV associated parasite antigens by MHC-I, wild type (WT) and Irgm1/m3 deficient (Irgm1/m3^−/−^) bone marrow derived macrophages (BMMΦs) or bone marrow derived dendritic cells (BMDCs) were primed with IFN-γ, or left unprimed and then host cells were infected with RH or RH-OVA. Irgm1/m3^−/−^ BMMΦs or BMDCs lack functional IRG effector molecules. Presentation of the OVA SIINFEKL peptide by MHC-I on the host cell surface was measured using the sensitive and quantitative B3Z antigen presentation assay (43) in previously optimized assay conditions to determine the fold change (or fold-activation) in antigen presentation in host cells infected with RH-OVA compared to host cells infected with parental RH not expressing OVA antigen (3, 13). As expected, RH infected WT or Irgm1/m3^−/−^ BMMΦs or BMDCs induced only low background levels of B3Z CD8^+^ T cell activation (<1-fold change). In contrast, antigen presentation by RH-OVA infected WT BMMΦs primed with IFN-γ was markedly increased in comparison to unprimed WT BMMΦs, unprimed Irgm1/m3^−/−^ BMMΦs, as well as primed Irgm1/m3^−/−^ BMMΦs infected with RH-OVA parasites (Figure 1, *top panel*). However, in contrast to macrophages, antigen presentation was increased in both IFN-γ primed WT BMDCs as well as in IFN-γ primed Irgm1/m3^−/−^ BMDCs (Figure 1, *bottom panel*). These results show that presentation of PV localized OVA antigen by MHC-I is IFN-γ and Irgm1/m3 dependent in macrophages, and IFN-γ dependent but Irgm1/m3 independent in dendritic cells. Thus, IFN-γ was required for stimulating antigen presentation in parasite infected cells, whereas the role of Irgm1/m3 molecules in antigen presentation of parasite antigens was cell type dependent.

### Development of Isogenic OVA-Expressing Parasite Strains Deleted for Parasite Rhoptry and Dense Granule Secreted Effectors

Since IFN-γ primed presentation of PV localized *Toxoplasma* antigens by MHC-I through both Irgm1/m3 dependent and Irgm1/m3 independent mechanisms (Figure 1), we hypothesized that the parasite may utilize a range of secreted effectors to limit antigen presentation by MHC-I. To address this hypothesis, we first identified candidate secreted *Toxoplasma* rhoptry (ROP) and dense granule (GRA) secreted proteins that have been previously localized to the PV, the PVM, the intravacuolar network (IVN) membranes within the PV lumen, or that have been linked to parasite resistance against host IFN-γ or IRG effectors. Knockouts of ROP5, ROP18, GRA2, GRA3, GRA4, GRA5, GRA6, GRA7, GRA8, and GRA12 were developed in the isogenic parental RH-OVA [Δ*ku80*] background. Several knockout strains were complemented with either the corresponding WT gene allele or with a gene allele possessing an engineered mutation (Figures S2, S3). All strains engineered in this study are listed in Table S1. OVA expression and secretion into the PV lumen was initially assessed by confocal microscopy, and as expected by virtue of sharing an identical single copy OVA gene, OVA accumulation in the PV appeared to be equivalent in each of these isogenic mutant strains (Figure S1D). In addition, we selectively examined the expression of PV localized OVA, compared to expression of PV localized GRA5, using a FACS based assay to verify that in a large population of *Toxoplasma*- infected macrophages (Figure S4A), or infected dendritic cells (Figure S4B), the expression of PV localized OVA was equivalent between the different isogenic mutant strains. Thus, under the antigen presentation assay conditions used here to measure the presentation of PV localized OVA antigen by MHC-I, the PVs between the different mutant strains expressed equivalent amounts of PV localized OVA antigen.

### ROP5 Regulates the Presentation of PV Localized OVA Antigen by MHC-I Through Irgm1/m3 Dependent and Irgm1/m3 Independent Mechanisms in Macrophages and Dendritic Cells

**Figure 2:**
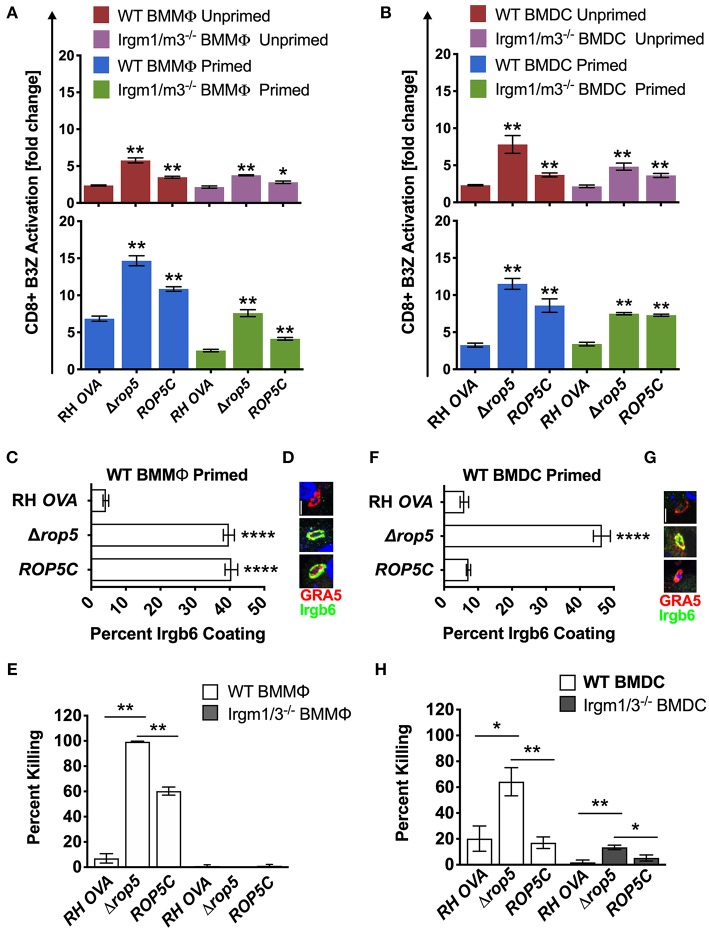
Δ*rop5* increases antigen presentation by BMMΦs and BMDCs and decreased survival in BMMΦs. Antigen presentation in WT and Irgm1/m3^−/−^ BMMΦs **(A)** and WT and Irgm1/m3^−/−^ BMDCs **(B)** infected with wild type OVA expressing parasites (RH-OVA), Δ*rop5* knockout (Δ*rop5)*, and complemented Δ*rop5* strain (*ROP5C*). For statistical analysis B3Z activation was compared between RH OVA and the specified parasite strain for the infecting APC (i.e., WT or Irgm1/m3^−/−^). Quantification **(C)** and immunofluorescence **(D)** of representative images of Irgb6 (green) and GRA5 (red) localization on the PV in BMMΦs. Comparison of *in vitro* killing of PVs **(E)** by primed and unprimed BMMΦs as determined by plaque forming units (PFUs) at day 6 post infection. Quantification **(F)** and immunofluorescence **(G)** representative images of Irgb6 (green) and GRA5 (red) localization on the PV in BMDCs. Comparison of *in vitro* killing of PVs **(H)** by primed and unprimed BMDCs as determined by PFUs at day 6 post infection. Mean ± SEM. Mann-Whitney test. *P < 0.05, **P < 0.01, ****P < 0.0001.

Given previous findings that Δ*rop5* PVs are easily physically disrupted by the action of Irgm1/m3 dependent host IRG effectors (20, 22, 23, 26, 48), we hypothesized that host cells infected with Δ*rop5* parasites may exhibit increased levels of antigen presentation by MHC-I molecules from the release of PV localized OVA antigen into the host cell cytosol following IRG-mediated destruction of the PV. To assess this hypothesis, we measured B3Z CD8^+^ T cell activation in unprimed or IFN-γ primed WT and Irgm1/m3^−/−^ BMMΦs and BMDCs infected with RH-OVA or Δ*rop5* parasites. Compared to RH-OVA, antigen presentation in cells infected with Δ*rop5* parasites was markedly increased in all conditions (Figure 2A). In contrast to antigen presentation by BMMΦs infected with parental RH-OVA that was dependent on both IFN-γ and Irgm1/m3, antigen presentation in BMMΦs infected with Δ*rop5* parasites was boosted by priming with IFN-γ, but was not strictly dependent on either IFN-γ or Irgm1/m3^−/−^ (Figure 2A). These results suggested that ROP5 controls two distinct pathways of antigen presentation by macrophages, one pathway is dependent on Irgm1/m3 molecules while the second pathway is independent of Irgm1/m3 molecules. In addition, IFN-γ priming of macrophages increases antigen presentation by both pathways. Remarkably, compared with BMMΦs, essentially identical patterns of Irgm1/m3 dependent and Irgm1/m3 independent antigen presentation were also observed in BMDCs infected with Δ*rop5* parasites (Figure 2B). Thus, the loss of ROP5 created identical patterns of antigen presentation in BMMΦs and BMDCs, whereas in cells infected with parental RH-OVA that expresses ROP5, antigen presentation by macrophages was dependent on Irgm1/m3 and IFN-γ, while antigen presentation by dendritic cells was dependent only on IFN-γ (Figures 1, 2).

The *ROP5* locus in the virulent type I RH strain appears to express 2 copies of the *ROP5A* gene allele type, 2 copies of the *ROP5B* gene allele type, and 2 copies of the *ROP5C* gene allele type (22). All six of these *ROP5* gene alleles were deleted in Δ*rop5* parasites. The *ROP5C* gene allele type was previously correlated with IRG evasion and virulence in mice (24). Therefore, we complemented Δ*rop5* parasites with a single copy of the *ROP5C* gene allele (Δ*rop5*::*ROP5C* parasites) (Figure S2). Expression of the *ROP5C* gene allele partially rescued the Irgm1/m3 and IFN-γ dependent antigen presentation phenotype in BMMΦs infected with Δ*rop5*::*ROP5C* parasites (Figure 2A). In contrast, antigen presentation levels were similar in WT and Irgm1/m3^−/−^ BMDCs infected with Δ*rop5*::*ROP5C* parasites (Figure 2B), suggesting that the *ROP5C* allele substantially rescued the Irgm1/m3 and IFN-γ dependent antigen presentation phenotype in dendritic cells. These results also suggested that the *ROP5C* gene allele did not substantially rescue the Irgm1/m3 independent antigen presentation phenotype in macrophages or in dendritic cells.

### ROP5 Regulates PV Survival in Macrophages and Dendritic Cells

To assess the role of Irgm1/m3 dependent IRG effectors and PV disruption in ROP5 mediated antigen presentation phenotypes, we measured IRG coating of the PVM and PV killing in IFN-γ primed BMMΦs and BMDCs infected with RH-OVA, Δ*rop5*, or Δ*rop5*::*ROP5C* parasites. Compared with parental RH-OVA, Irgb6 coating of the PVM was strikingly increased in IFN-γ primed WT BMMΦs (Figures 2C,D) and in IFN-γ primed WT BMDCs (Figures 2F,G) infected with Δ*rop5* parasites. BMDCs infected with Δ*rop5* parasites expressing a single *ROP5C* gene allele (Δ*rop5*::*ROP5C* parasites) (Figure S2) resisted Irgb6 coating of the PVM as well as the parental RH-OVA strain (Figures 2F,G). In contrast, resistance to Irgb6 coating of the PVM was not rescued in BMMΦs infected with Δ*rop5*::*ROP5C* parasites (Figures 2C,D). As expected, PV survival was not detected in WT BMMΦs infected with Δ*rop5* parasites (Figure 2E), though PV survival was partially rescued in WT BMMΦs infected with Δ*rop5*::*ROP5C* parasites (Figure 2E). These results suggested that PV survival in IFN-γ primed macrophages required multiple *ROP5* alleles to resist the IFN-γ and Irgm1/m3 dependent mechanisms of PV killing (Figure 2E), though a single *ROP5C* allele was sufficient for PV survival in dendritic cells (Figure 2H). In addition, these phenotypes correlated with reduced killing (~60%) of PVs in dendritic cells compared to killing (~100%) of PVs in macrophages infected with Δ*rop5* parasites. Compared to parental RH-OVA, killing of PVs was increased only ~3-fold in BMDCs infected with Δ*rop5* parasites, whereas killing of PVs was increased at least 20-fold in BMMΦs infected with Δ*rop5* parasites (Figures 2E,H). While PV killing was not detected in Irgm1/m3^−/−^ BMMΦs infected with Δ*rop5* parasites (Figure 2E), surprisingly, we found that ROP5 also regulated an Irgm1/m3 independent mechanism of PV killing in IFN-γ primed BMDCs (Figure 2H). Moreover, a single *ROP5C* gene allele rescued PV survival in IFN-γ primed Irgm1/m3^−/−^ BMDCs infected with Δ*rop5*::*ROP5C* parasites (Figure 2H). Consistent with these *ex vivo* PV killing phenotypes, complementation of Δ*rop5* parasites with the *ROP5C* gene allele significantly rescued the acute virulence of Δ*rop5* parasites in mice (Figure S5). Collectively, these findings suggest that ROP5 limited antigen presentation in macrophages and dendritic cells by neutralizing Irgm1/m3 and IFN-γ dependent host IRG effectors to prevent PV disruption and the release of PV localized OVA antigen for presentation by host MHC-I molecules. ROP5 also blocked an Irgm1/m3 independent pathway for the presentation of PV localized antigens by MHC-I. This Irgm1/m3 independent pathway was boosted by priming host cells with IFN-γ, but was not significantly associated with PV clearance in macrophages or dendritic cells.

### ROP18 Regulates Antigen Presentation Primarily Through Irgm1/m3 Dependent Mechanisms in Macrophages and Through Irgm1/m3 Independent Mechanisms in Dendritic Cells

**Figure 3:**
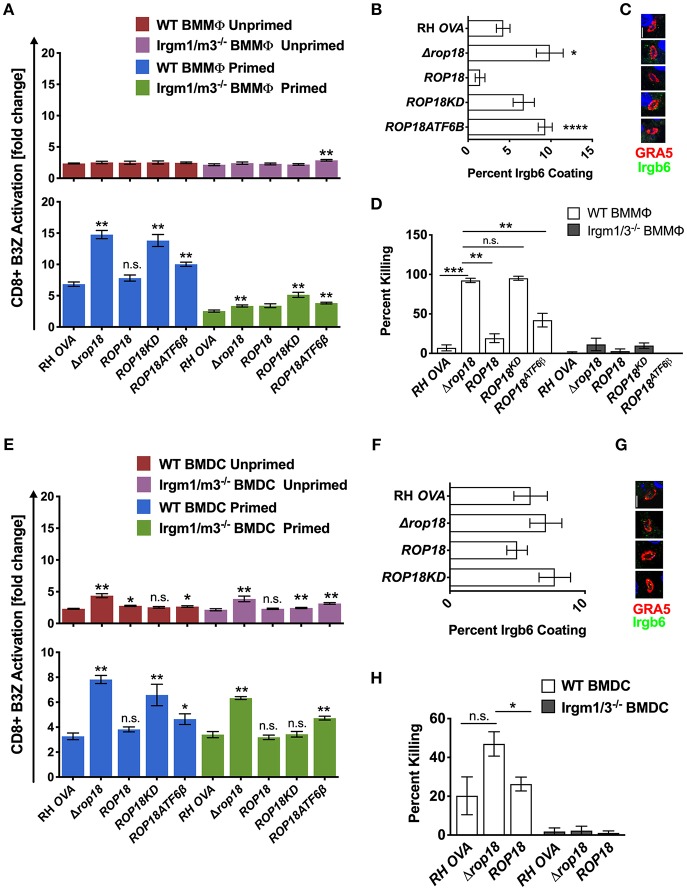
Infection with the Δ*rop18* strain results in increased antigen presentation by BMMΦs and BMDCs corresponding with decreased parasite survival. Antigen presentation in WT and Irgm1/m3^−/−^ BMMΦs **(A)** or WT and Irgm1/m3^−/−^ BMDCs **(E)** infected with wild type OVA expressing parasites (RH-OVA), Δ*rop18* knockout (Δ*rop18)*, wild-type complemented Δ*rop18::ROP18* strain (*ROP18*), kinase-dead (inactive) complemented Δ*rop18::ROP18*^*KD*^ (*ROP18KD*), and Δ*rop18*::*ROP18*^*ATF*6β^ (*ROP18ATF6*β). For statistical analysis B3Z activation was compared between RH OVA and the specified parasite strain for the infecting APC (i.e., WT or Irgm1/m3^−/−^). Quantification **(B)** and immunofluorescence **(C)** representative images of Irgb6 (green) and GRA5 (red) localization on the PV in BMMΦs. Comparison of fold change in percent killing of PVs between knockout and RH-OVA **(D)** by primed and unprimed BMMΦs as determined by PFUs at day 6 post infection. Quantification **(F)** and immunofluorescence **(G)** representative images of Irgb6 (green) and GRA5 (red) localization on the PV in BMDCs. Comparison of *the* fold change in percent killing of PVs between knockout and RH-OVA **(H)** by primed and unprimed BMDCs as determined by PFUs at day 6 post infection. Mean ± SEM. Mann-Whitney test. *P < 0.05, **P < 0.01, ***P < 0.001, ****P < 0.0001.

ROP5 is a central component of distinct ROP17 or ROP18 high molecular weight PVM associated protein complexes (25) where it regulates the ability of ROP18 to phosphorylate IRGs (23) to neutralize their PV killing effector functions (36). In contrast to Δ*rop5* parasites (Figure 2A, *top panel*), antigen presentation was not increased in unprimed WT or Irgm1/m3^−/−^ BMMΦs infected with Δ*rop18* parasites (Figure 3A, *top panel*). However, IFN-γ primed WT BMMΦs infected with Δ*rop18* parasites exhibited markedly increased antigen presentation compared to IFN-γ primed Irgm1/m3^−/−^ BMMΦs (Figure 3A, *bottom panel*). Together, these results suggested that ROP18 selectively suppressed an Irgm1/m3 and IFN-γ dependent pathway of antigen presentation in macrophages. Consistent with these results, Irgb6 coating of the PVM (Figures 3B,C) and PV killing (Figure 3D) was increased in IFN-γ primed WT BMMΦs infected with Δ*rop18* parasites. Complementation of Δ*rop18* parasites with the WT *ROP18* gene allele (Δ*rop18*::*ROP18)* (Figure S3) rescued these Irgm1/m3 and IFN-γ dependent antigen presentation, Irgb6 coating, and PV killing phenotypes (Figures 3A–D). In contrast, complementation of Δ*rop18* parasites with ROP18 mutants possessing a non-functional kinase-dead (KD) domain (*ROP18KD*) that fails to phosphorylate IRGs (36), or with a ROP18 mutant with a deletion of the N-terminal ATFβ6 domain (*ROP18ATF6*β) (Figure S3) that mediates ROP18 association to the host ATFβ6 sensor (37), as well as to the PVM (49, 50), failed to fully rescue these phenotypes (Figures 3A–D). Thus, the Irgm1/m3 and IFN-γ dependent ROP18 mediated suppression of antigen presentation in macrophages was dependent on the ROP18 kinase activity and the N-terminal ATFβ6 domain of ROP18. Collectively, these results suggested that ROP18 limited antigen presentation in IFN-γ primed macrophages primarily through its Irgm1/m3 dependent interaction with IRG effector proteins to prevent PV disruption and the release of PV localized OVA antigen.

In contrast to unprimed WT and Irgm1/m3^−/−^ macrophages infected with Δ*rop18* parasites, a significant increase in antigen presentation was observed in unprimed WT and Irgm1/m3^−/−^ BMDCs infected with Δ*rop18* parasites (Figure 3E, *top panel*). Moreover, in contrast to IFN-γ primed WT and Irgm1/m3^−/−^ macrophages (Figure 3A, *bottom panel*), the levels of antigen presentation were similar between IFN-γ primed WT and Irgm1/m3^−/−^ BMDCs infected with Δ*rop18* parasites (Figure 3E, *bottom panel*). This Irgm1/m3 independent pathway of increased antigen presentation in BMDCs infected with Δ*rop18* parasites was rescued by the WT allele of *ROP18* (Δ*rop18*::*ROP18*) (Figure 3E, *bottom panel*). Together, these results suggested that ROP18 suppressed antigen presentation in dendritic cells primarily through a mechanism that was not dependent on Irgm1/m3 molecules. Consistent with these results, the absence of ROP18 did not affect Irgb6 coating of the PVM in BMDCs (Figures 3F,G) and only weakly influenced PV killing (Figure 3H). In addition, ROP18's ability to suppress this Irgm1/m3 independent pathway of antigen presentation was significantly rescued in unprimed as well as in IFN-γ primed Irgm1/m3^−/−^ BMDCs infected with kinase-dead Δ*rop18*::*ROP18KD* parasites, or infected with Δ*rop18*::*ROP18ATF6*β parasites (Figure 3E). These results suggested that the kinase activity and the ATF6β domain of ROP18 were not strictly necessary for ROP18's ability to suppress the Irgm1/m3 independent mechanism of antigen presentation in dendritic cells.

### PVM Associated Dense Granule Proteins GRA3 and GRA7 Regulate Antigen Presentation

**Figure 4:**
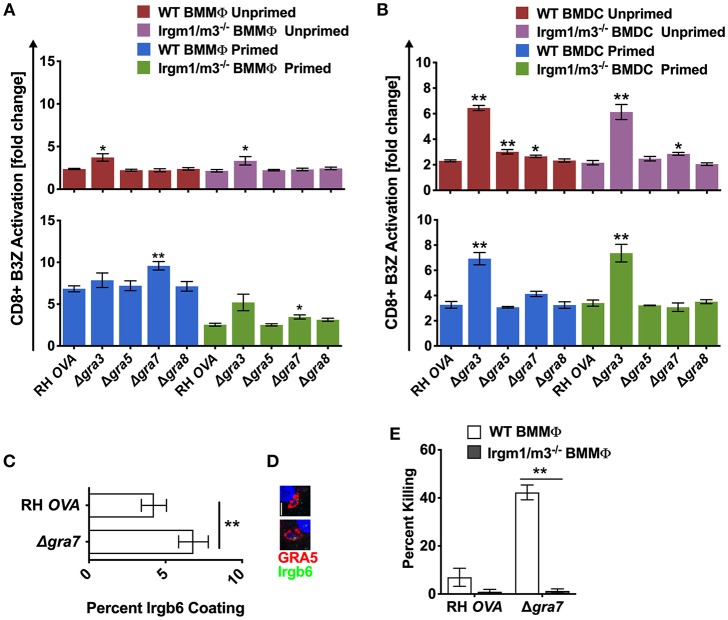
Δ*gra3* exhibits BMDC specific and IRG and IFN-γ independent increases in antigen presentation. Antigen presentation in WT and Irgm1/m3^−/−^ BMMΦs **(A)** and WT and Irgm1/m3^−/−^ BMDCs **(B)** infected with wild type OVA expressing parasites (RH-OVA), Δ*gra3*, Δ*gra5*, Δ*gra7, or* Δ*gra8*. Quantification **(C)** and immunofluorescence **(D)** representative images of Irgb6 (green) and GRA5 (red) localization on the PV in BMMΦs. Comparison of fold change in percent killing of PVs between knockout and RH-OVA **(E)** by primed and unprimed BMMΦs as determined by PFUs at day 6 post infection. For statistical analysis B3Z activation was compared between RH *OVA* and Δ*gra3* strains in the specified host cells. Mean ± SEM. Mann-Whitney test. *P < 0.05, **P < 0.01.

To determine whether other PVM localized proteins also influenced antigen presentation, we evaluated the role GRA3, GRA5, and GRA8, three other secreted dense granule proteins that selectively localize to the PVM (51). GRA8 played no detectable role in regulating antigen presentation in macrophages or dendritic cells (Figures 4A,B). Antigen presentation was selectively, and slightly, increased in unprimed WT BMDC infected with Δ*gra5* parasites (Figure 4A). In contrast, antigen presentation was markedly increased in unprimed and in IFN-γ primed WT and Irgm1/m3^−/−^ BMMΦs and BMDCs infected with Δ*gra3* parasites (Figures 4A,B). Remarkably, the increases in antigen presentation in unprimed and in IFN-γ primed WT and Irgm1/m3^−/−^ BMDCs infected with Δ*gra3* parasites were similar in magnitude (Figure 4B). These results suggested that GRA3 regulated a mechanism of antigen presentation that was not specifically dependent on Irgm1/m3 molecules, IFN-γ, or cell type.

The PVM localized GRA7 molecule associates with ROP5/ROP18 high molecular weight protein complexes (25). Consistent with this localization, GRA7 was previously shown to coordinate its activities with the ROP18 kinase to enhance resistance to IRG mediated clearance in macrophages (52, 53). Consistent with this role for GRA7 and our hypothesis that PV disruption increases antigen presentation of PV localized antigens by MHC-I, antigen presentation was unaffected in unprimed BMMΦs and was markedly increased in IFN-γ primed WT BMMΦs, indicating that GRA7 suppressed a major IFN-γ and IRG dependent pathway of antigen presentation in macrophages. This GRA7 phenotype in BMMΦs was similar to, though slightly reduced in magnitude, in comparison to BMMΦs infected with Δ*rop18* parasites (Figure 3A). In addition, minor increases in antigen presentation were observed in primed Irgm1/m3^−/−^ BMMΦs (Figure 4A), and in unprimed Irgm1/m3^−/−^ BMDCs (Figure 4B) infected with Δ*gra7* parasites. As expected, Δ*gra7* PVs exhibited increased coating with Irgb6 in IFN-γ primed BMMΦs (Figures 4C,D), and consistent with this observation, increased Irgm1/m3 dependent killing of the Δ*gra7* PV (Figure 4E). Collectively, these results suggested that GRA7 limited antigen presentation primarily in IFN-γ primed macrophages through its Irgm1/m3 dependent interaction with IRG effectors to prevent PV disruption and the release of PV localized OVA antigen for presentation by MHC-I.

### Intravacuolar Network Membrane Associated GRA Proteins Regulate Antigen Presentation

**Figure 5:**
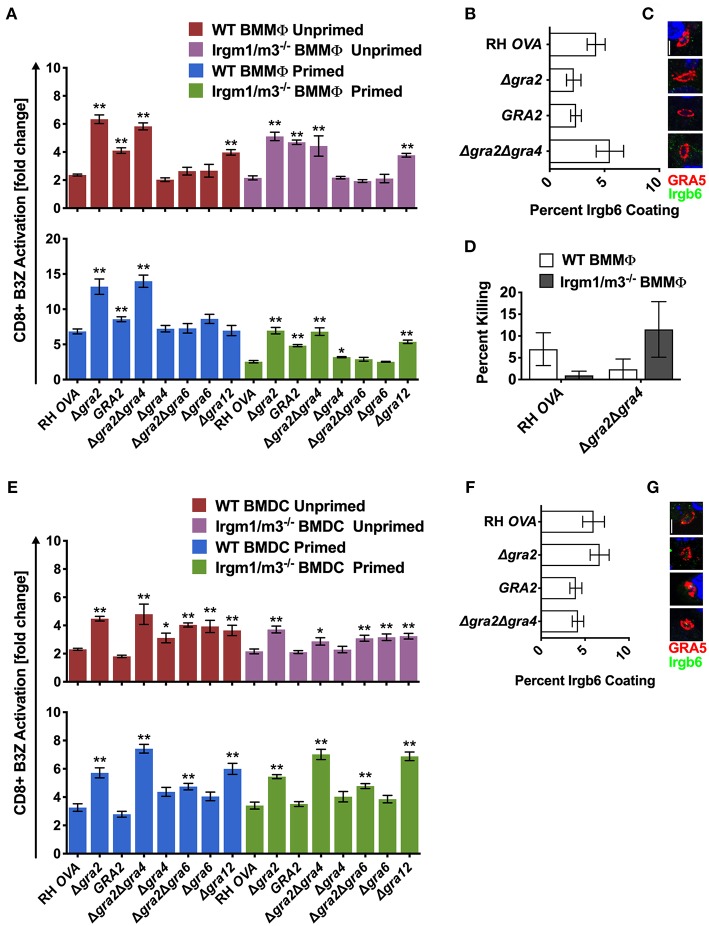
Δ*gra2* increases antigen presentation in BMMΦs in the presence of GRA6. Antigen presentation in WT and Irgm1/m3^−/−^ BMMΦs **(A)** or WT and Irgm1/m3^−/−^ BMDCs **(E)** infected with wild type OVA expressing parasites (RH-OVA), Δ*gra2* knockout (Δ*gra2)*, wild-type complemented Δ*gra2::GRA2* strain (*GRA2*), Δ*gra2*Δ*gra4* double knockout (Δ*gra2*Δ*gra4)*, Δ*gra4* knockout (Δ*gra4)*, Δ*gra2*Δ*gra6* double knockout (Δ*gra2*Δ*gra6)*, Δ*gra6* knockout (Δ*gra6)*, Δ*gra12* knockout (Δ*gra12)*. For statistical analysis B3Z activation was compared between RH OVA and the specified parasite strain for the infecting APC (i.e., WT or Irgm1/m3^−/−^). Quantification **(B)** and immunofluorescence **(C)** representative images of Irgb6 (green) and GRA5 (red) localization on the PV in BMMΦs. Comparison of fold change in percent killing of PVs between knockout and RH-OVA **(D)** by primed and unprimed BMMΦs as determined by PFUs at day 6 post infection. Quantification **(F)** and immunofluorescence **(G)** of representative images showing Irgb6 (green) and GRA5 (red) localization on the PV in BMDCs. Mean ± SEM. Mann-Whitney test. *P < 0.05, **P < 0.01.

In addition to GRA7, the GRA12 molecule was also previously shown to be selectively associated with high molecular weight ROP5 and ROP18 PVM localized protein complexes (25). Moreover, GRA12 was previously localized to a nanotubular membrane system in the PV (54) that is called the nanotubular membranous network, or alternatively, the intravacuolar network (IVN). GRA2, GRA4, GRA6, and GRA12 localize to the membranes that comprise the intravacuolar network (IVN) (51). GRA6 is required for the maintenance of IVN membrane structures in the PV, while GRA2 is required for the assembly of the membranous nanotubules that comprise the IVN (55). GRA2 was previously reported to influence IRG association with the PVM association (24, 52), and GRA2 has also been reported to suppress the presentation of the endogenous membrane-bound GRA6 C-terminal epitope by MHC-I (42). To examine the role of IVN localized GRA proteins in regulating antigen presentation of the soluble OVA PV localized antigen, we engineered several isogenic OVA-expressing GRA knockout strains (Δ*gra2*, Δ*gra2*::GRA2, Δ*gra2*Δ*gra4*, Δ*gra2*Δ*gra6*, Δ*gra4*, Δ*gra6*, and Δ*gra12*) (Figure S1). Antigen presentation was increased in unprimed and IFN-γ primed WT and Irgm1/m3^−/−^ BMMΦs and BMDCs infected with Δgra12 parasites. Antigen presentation levels in unprimed or IFN-γ primed WT and unprimed or IFN-γ primed Irgm1/m3^−/−^ BMMΦs and BMDCs infected with Δ*gra12* parasites were similar (Figures 5A,E). These results suggested that GRA12 regulated a mechanism of antigen presentation that was not dependent on Irgm1/m3 molecules, IFN-γ, or cell type.

Similar and significant increases in antigen presentation were observed in unprimed and IFN-γ primed WT or Irgm1/m3^−/−^ BMMΦs infected with Δ*gra2* or Δ*gra2*Δ*gra4* parasites (Figure 5A). In addition, increased antigen presentation was observed in IFN-γ primed WT BMMΦs compared with IFN-γ primed Irgm1/m3^−/−^ BMMΦs infected with Δ*gra2* or Δ*gra2*Δ*gra4* parasites (Figure 5A). In contrast, no increase in antigen presentation was observed in BMMΦs infected with Δ*gra4* parasites (Figure 5A), suggesting that it was the absence of GRA2 that was most important for the antigen presentation phenotype in BMMΦs infected with Δ*gra2*Δ*gra4* parasites. Complementation of Δ*gra2* parasites with the WT *GRA2* gene allele inserted into the *UPRT* locus significantly rescued these Δ*gra2* antigen presentation phenotypes in BMMΦs (Figure 5A). These results suggested that GRA2 regulated Irgm1/m3^−/−^ dependent and Irgm1/m3 independent mechanisms of antigen presentation in BMMΦs infected by Δ*gra2* or Δ*gra2*Δ*gra4* parasites. However, in contrast to macrophages infected with Δ*rop5* (Figures 2C–E), Δ*rop18* (Figures 3B–D), or Δ*gra7* parasites (Figure 4E), increased Irgb6 coating of the PVM (Figures 5B,C) or increased PV killing were not observed in IFN-γ primed WT BMMΦs infected with Δ*gra2*Δ*gra4* parasites (Figure 5D). These results suggested that deletion of GRA2 increased Irgm1/m3 and IFN-γ dependent antigen presentation in BMMΦs without any associated increase in IRG mediated PV clearance.

Antigen presentation levels were similar between IFN-γ primed WT BMDCs and IFN-γ primed Irgm1/m3^−/−^ BMDCs infected with Δ*gra2* or Δ*gra2*Δ*gra4* parasites (Figure 5E). In contrast, only small increases in antigen presentation were observed in BMDCs infected with Δ*gra4* parasites (Figure 5E), suggesting that it was the absence of GRA2 that was necessary for the antigen presentation phenotype in BMDCs infected with Δ*gra2*Δ*gra4* parasites. These results suggest that GRA2 regulated an Irgm1/m3 independent mechanism of antigen presentation in dendritic cells. Complementation of Δ*gra2* parasites with the WT *GRA2* gene allele rescued the Irgm1/m3 independent antigen presentation phenotype in BMDCs (Figure 5E). No increase in Irgb6 coating of the PVM was observed in BMDCs infected with Δ*gra2* or Δ*gra2*Δ*gra4* parasites (Figures 5F,G).

GRA2 was previously reported to limit CD8^+^ T cell recognition of the membrane-bound immunodominant HF10 epitope present at the C-terminus of the GRA6 antigen (42). This interpretation of GRA2-associated antigen presentation phenotypes was based on the assumption that the GRA6 molecule itself did not influence antigen presentation by MHC-I (42). Thus, to formally eliminate any potential role of the GRA6 molecule in affecting the antigen presentation phenotypes of mutants deleted for GRA2, we developed Δ*gra6* parasites as well as double knockout Δ*gra2*Δ*gra6* parasites (Figure S1). No significant antigen presentation phenotype was observed in BMMΦs infected with Δ*gra6* parasites (Figure 5A), though antigen presentation was slightly increased in unprimed or IFN-γ primed WT or Irgm1/m3^−/−^ BMDCs infected with Δ*gra6* parasites (Figure 5E). However, the absence of both GRA6 and GRA2 molecules in Δ*gra2*Δ*gra6* parasites markedly decreased or abolished the antigen presentation phenotypes observed in BMMΦs and BMDCs infected with Δ*gra2* or Δ*gra2*Δ*gra4* parasites (Figures 5A,E). These results revealed that the expression of the GRA6 protein was required to observe the major increase in antigen presentation by MHC-I in host cells infected with Δ*gra2* parasite strains.

## Discussion

**Figure 6:**
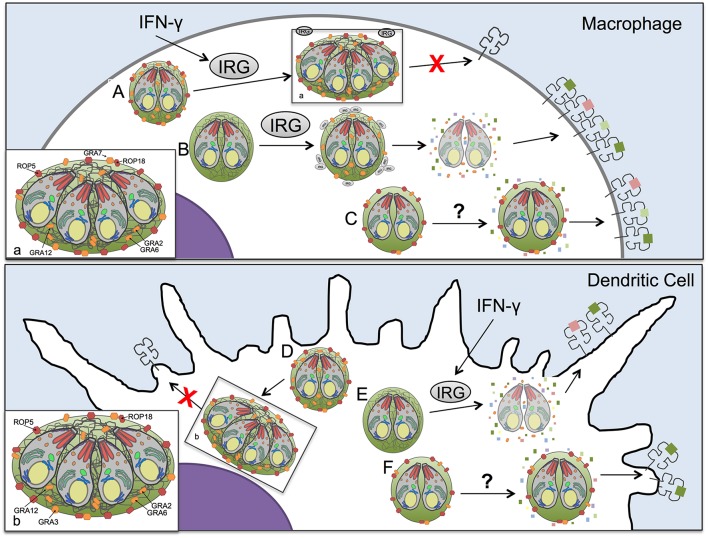
Model of antigen presentation by MHC-I in *Toxoplasma* infected antigen presenting cells. **(A)** In *Toxoplasma* infected WT macrophages low levels of antigen presentation occur in an IFN-γ and IRG-dependent manner, with minimal Irgb6 vacuole coating and parasite killing. **(B)** However, in macrophages, if the parasites lack ROP5 or ROP18 molecules that can resist IRGs, the IRG proteins are able to coat the vacuole and disrupt the PV, releasing antigen into the host cell cytosol for increased processing and presentation by MHC-I. Yet as we demonstrate in this study, IRGs are not the only mechanism by which antigen is released from the parasitophorous vacuole (PV) for antigen presentation. **(C)** In the absence of IRGs and IFN-γ stimulation macrophages infected with the Δ*gra2* knockout that lacks a mature intravacuolar network (IVN) increased antigen presentation selectively in the presence of GRA6 but not in the absence of GRA6, highlighting the importance of IVN associated GRA proteins in regulating presentation of PV antigens. Additionally, another IVN localized GRA protein, GRA12, suppresses antigen presentation in macrophages independently of IFN-γ or IRGs. **(D)** A parasite that expresses ROP5, ROP18, GRA2, and GRA3 has minimal levels of antigen presentation in dendritic cells. **(E)** If the dendritic cell is activated with IFN-γ it activates IRGs that lead to disruption of the parasitophorous vacuole of parasites that lack ROP5 or ROP18 molecules that can resist IRGs thereby increasing antigen presentation. **(F)** The loss of GRA2, which leads to an absence of intravacuolar network, increases antigen presentation in dendritic cell selectively in the presence of GRA6. As well, there appears to be dendritic cell-specific mechanisms for accessing antigens in the parasitophorous vacuole, as only in dendritic cells does the loss of GRA3 increase antigen presentation in the absence of IRGs and IFN-γ stimulation.

The CD8^+^ T cell response is essential for establishing control of *Toxoplasma* infection (56, 57), yet the mechanisms by which *Toxoplasma* antigens enter the host cell cytosol for presentation by MHC-I and whether secreted *Toxoplasma* effector proteins regulate antigen presentation are unknown (15). Here we demonstrate that host IFN-γ and IRG effector proteins contribute to antigen presentation in macrophages as well as in dendritic cells and we find that parasite antigens released from disrupted parasite vacuoles are presented by host cell MHC-I molecules. Additionally, our results suggest that *Toxoplasma* secreted effectors, ROP5, ROP18, GRA2, GRA3, GRA7, and GRA12 suppressed antigen presentation by infected host cells (Figure S6). The diversity of parasite molecules capable of limiting presentation of PV localized antigens in both the presence and absence of IRG proteins, or stimulation of cells by IFN-γ, suggests a multi-layered system for presentation within cells and distinct mechanisms between macrophages and dendritic cells. Our results highlight the adaptability of the parasite to multiple cellular environments and the necessity of parasite control of host cell antigen presentation mechanisms within infected cells, which are regulated by the secretion of distinct ROP and GRA proteins. Remarkably, the GRA2 antigen presentation phenotype was found to be co-dependent on expression of the GRA6 molecule. Our work underscores that *Toxoplasma* deploys multiple mechanisms mediated by rhoptry and dense granule secreted effectors associated with the PVM and the IVN membrane systems to regulate IRG effector dependent and independent mechanisms that control the presentation of soluble parasite PV localized antigens by MHC-I in infected host cells for CD8^+^ T cell recognition (Figure 6).

It is well-established that the immunodominant antigens of *Toxoplasma* recognized by CD8^+^ T cells are associated with the PV (12, 13). Moreover, the development of a protective CD8^+^ T cell response to *Toxoplasma* is dependent on parasite invasion and PV formation within myeloid cells (5). Since *Toxoplasma* infection prevents apoptosis of the host cell, this process of antigen presentation by MHC-I in cells invaded by *Toxoplasma* is not dependent upon apoptosis of the host cell (58–60). Thus, the PVM serves as a barrier to host cell access to relevant parasite antigens in the PV. Previous studies reported that PV localized antigens were optimally recognized by CD8^+^ T cells if the antigen was localized to the lumen of the PV (12, 13). Moreover, IRG proteins were previously shown to enhance antigen presentation of type II *Toxoplasma* in macrophages (3) as well as in mouse embryonic fibroblasts (61) suggesting that disruption of the PVM by host IRG effectors (15, 16, 62) increased host cell access to PV localized antigens and their presentation by MHC-I molecules. Our results confirmed these previous findings and show that antigen presentation of soluble PV lumen localized antigen by MHC-I is dependent on IFN-γ stimulation and functional IRG effector molecules in macrophages infected with the virulent type I RH strain. In infected dendritic cells, however, antigen presentation was not dependent on IRG effectors but was increased by IFN-γ stimulation, as well as PV killing. Indeed, our study is the first to examine a role for IRG dependent killing on the *Toxoplasma* PV in dendritic cells. These findings suggested that parasite secreted effectors regulated IRG dependent and independent pathways of antigen presentation in host cells infected with the virulent type I RH strain.

Rhoptry proteins ROP5 and ROP18 block host IRG's to preserve the integrity of the PVM (20, 22–24, 26, 36, 38, 48, 49, 63, 64). Remarkably, essentially identical patterns of IRG effector dependent and independent antigen presentation were observed in macrophages and dendritic cells infected with Δ*rop5* parasites, suggesting that ROP5 suppressed both pathways of antigen presentation. The Irgm1/m3 and IFN-γ dependent pathway in macrophages and dendritic cells correlated with increased Irgb6 coating of the PVM and destruction of the PV, though macrophages were more efficient in killing PVs than dendritic cells. Our results show that ROP5 blocked components of the IRG effector pathway in both macrophages and dendritic cells, but ROP5 did not block the ability of IFN-γ to stimulate antigen presentation in infected host cells. Thus, ROP5 blocked the IFN-γ and Irgm1/m3 dependent pathway of antigen presentation by neutralizing IRG effectors to preserve the integrity of the PVM and prevent PV disruption and PV clearance. These findings suggest that PV antigens released from damaged, or killed, PVs were more efficiently presented by MHC-I than PV antigens in intact PVs.

In addition to the ability of ROP5 to prevent IRG and IFN-γ mediated PV killing in macrophages and dendritic cells, our data also revealed that ROP5 prevented PV killing in IFN-γ activated Irgm1/m3 deficient dendritic cells. This IFN-γ dependent and IRG independent pathway of PV killing was detected in dendritic cells, however, this pathway was not observed in macrophages. These results suggest that dendritic cells can use both IRG dependent and IRG independent mechanisms to kill and clear PVs in infected cells. In addition to IRG effectors, IFN-γ regulates host cell guanylate binding proteins (GBPs) and autophagy proteins that also associate with the PVM (47, 65–70). GBPs restrict PVs and perturb the PVM (47, 65–70). The targeting of IRG effectors as well as GBPs to the *Toxoplasma* PV is dependent on the expression of Irgm1/m3 molecules (47). In addition, ubiquitination of the PV is linked with the targeting of host p62 to the PV (71, 72). Colocalization of ubiquitin and p62 on *Toxoplasma* PVs was dependent on p62 stimulation with IFN-γ, particular autophagy proteins such as Atg3, Atg5, Atg7, and Atg16L1, and Irgm1/m3 molecules (73). Irgm1/m3 molecules regulate IRG and GBP targeting to the PV as well as p62 mediated ubiquitination of the PV (74). Moreover, PV ubiquitination and GBP targeting to the PV is dependent on initial IRG effector targeting of the PV (74). This PV ubiquitination pathway is regulated by p62 and the E3 ligase TRAF6 and PV ubiquitination licenses PVs for GBP binding (74). Consequently, ROP5 neutralization of IRG effectors also prevents PV ubiquitination and GBP targeting. Interestingly, host cell p62 was previously reported to play a role in the IFN-γ induced presentation of PV localized antigen without affecting cell autonomous clearance of PVs or the replication of parasites inside of PVs (73). Our experiments did not specifically investigate the role of GBPs, host autophagy proteins, PV ubiquitination, TRAF6, or P62 in the presentation of PV antigens by MHC-I and it remains to be determined to what extent these pathways contribute to Irgm1/m3 and IFN-γ dependent antigen presentation of PV localized antigens in infected macrophages and dendritic cells. Nonetheless, our results show that these pathways are not predicted to be involved in the Irgm1/m3 independent pathway of antigen presentation, or in the IFN-γ stimulated mechanisms that increase Irgm1/m3 independent antigen presentation.

While ROP5 regulated IRG effector dependent mechanisms of antigen presentation in macrophages and dendritic cells, ROP18 selectively regulated the IRG effector and IFN-γ dependent mechanism of antigen presentation in macrophages. ROP18 mediated suppression of antigen presentation in macrophages was dependent on the ROP18 kinase activity and the N-terminal ATFβ6 domain of ROP18, which is required for PVM association (49, 50). Thus, ROP18 limited antigen presentation in IFN-γ primed macrophages primarily through its Irgm1/m3 dependent interaction with IRG effectors to prevent PV disruption and the release of PV localized antigens. In addition to the role of ROP18 in targeting IRG effectors, the host cell NF-κB p65 molecule was also recently reported to be a phosphorylation target of ROP18, leading to the blockade of NF-κB activation (75). Since NF-κB can regulate MHC-I antigen presentation, this ROP18 function represents another potential mechanism to influence the IRG effector dependent pathway of antigen presentation in macrophages, and additional studies are still necessary to determine if ROP18 manipulation of NF-κB regulates antigen presentation by MHC-I.

ROP18 selectively suppressed the IRG effector independent pathway of antigen presentation in dendritic cells but not in macrophages. This finding contrasts with ROP5, which suppressed this pathway of antigen presentation in both macrophages and in dendritic cells. The ROP18 kinase activity was not required for suppressing antigen presentation in Irgm1/m3^−/−^ dendritic cells, though the ATFβ6 domain of ROP18 was partially required for this function. Collectively, our results suggest that ROP5 and ROP18 limited IRG effector independent antigen presentation in dendritic cells, while ROP5 alone was sufficient to limit this pathway in macrophages. In contrast, the major pathway of antigen presentation in macrophages is associated with IRG effector molecules and IFN-γ stimulation. ROP5 and ROP18 limited this pathway of antigen presentation by targeting and neutralizing IRG effectors.

ROP5 associates with ROP18, or independently with ROP17, to establish high molecular weight PVM associated protein complexes (25). The GRA7 molecule is a PVM associated molecule that was previously reported to selectively associate with ROP5/ROP18 protein complexes (25). GRA7 coordinates its activities to synergize with ROP18 to mediate more effective resistance to host IRG effectors (52, 53). Our results show that GRA7 suppressed an Irgm1/m3 and IFN-γ dependent pathway of antigen presentation selectively in macrophages. Moreover, we show that GRA7 was required for full resistance to PV clearance in a 6-day PFU assay, consistent with the recognized role of GRA7 in resisting IRG mediated PVM disruption in macrophages. However, previous data has indicated that the Δ*gra7* strain was not cleared by IFN-γ stimulated RAW macrophages in a 20-h assay (52). Together, these observations raise the question of whether complete disruption of the PVM is actually necessary for increased antigen presentation in macrophages, or whether PVM perturbations without PV clearance, is sufficient. Moreover, GRA7 was recently reported to induce TRAF6 activation through its physical association with TRAF6 via MyD88, thereby promoting more effective T cell immunity against *Toxoplasma* (76). Since GRA7 directly activates host innate immunity via MyD88 to activate TRAF6 (76), it is possible that deletion of GRA7 also results in reduced activation of TRAF6 and therefore reduced p62 mediated PV ubiquitination and GBP targeting of the PVM.

GRA3, GRA5, and GRA8 are other PVM associated molecules; however, deletion of GRA8 did not affect antigen presentation in macrophages or dendritic cells. GRA5 deletion was associated with a small increase in antigen presentation selectively in dendritic cells that was dependent on Irgm1/m3 but was not dependent on IFN-γ priming, hinting that Irgm1/m3, and IRGs, regulate a minor pathway of antigen presentation that is not linked to destruction of the PV by IRG effectors. In contrast, GRA3 regulated a significant pathway of antigen presentation. While antigen presentation trended slightly higher in dendritic cells than in macrophages infected with Δ*gra3* parasites, remarkably, the GRA3 antigen presentation phenotype was not critically dependent on Irgm1/m3 molecules, IFN-γ, or cell type. GRA3 is localized to the PVM and was previously predicted to interact with the host ER protein CAMLG (77, 78). It remains to be determined whether GRA3 interacts with other host specific molecules such as Rab22a or Sec22b that play a role in antigen presentation of PV localized antigens (79, 80), or whether vacuoles that lack GRA3 are simply leaky and directly release increased amounts of parasite antigens, or parasite molecules that influence antigen presentation by MHC-I.

Similar to ROP5 or ROP18 deletion, a significant IRG effector and IFN-γ dependent increase in antigen presentation was observed in macrophages infected with Δ*gra2* parasites. GRA2 selectively associates with the IVN membranes and was previously reported to influence IRG-PVM association (24, 81). However, in comparison to strains deleted for ROP18 or ROP5, Δ*gra2* parasites exhibit only a mild defect in acute virulence (82, 83). Moreover, in contrast to deletion of ROP5, ROP18, or GRA7, the IRG and IFN-γ dependent increase in antigen presentation in Δ*gra2* parasites did not correlate with any detectable changes in Irgb6 coating or PV clearance in IFN-γ stimulated macrophages. GRA2 limited antigen presentation by mechanisms that appear to be independent of rhoptry proteins.

GRA2 is necessary for normal formation of the membranous nanotubules of the IVN, and GRA6 also influences the IVN membrane structures (55, 84). While Δ*gra2*Δ*gra4* parasites exhibited significant and similar antigen presentation phenotypes as also observed in Δ*gra2* parasites, Δ*gra4*, Δ*gra6*, and surprisingly Δ*gra2*Δ*gra6* parasites closely resembled parental RH-OVA parasites and did not exhibit the major IRG and IFN-γ dependent increase in antigen presentation observed in macrophages, or the significant increases in antigen presentation in unprimed or IFN-γ primed Irgm1/m3^−/−^ macrophages or in unprimed or IFN-γ primed WT or Irgm1/m3^−/−^ BMDCs infected with Δ*gra2* or Δ*gra2*Δ*gra4* parasites. Consequently, the marked increase in IRG and IFN-γ dependent antigen presentation observed in macrophages, or in dendritic cells, infected with Δ*gra2* parasites was dependent on the expression of the GRA6 molecule. Remarkably, the Δ*gra2* IRG independent pathway of antigen presentation also required the expression of the GRA6 molecule. Thus, the major GRA2 antigen presentation phenotypes were dependent on the expression of the GRA6 protein. These results are striking and suggest an alternative model that may underpin the IVN associated mechanisms that control antigen presentation in parasite infected host cells.

GRA2 associates with other GRA proteins immediately following invasion and secretion of dense granules into the PV lumen while they are soluble, and upon membrane insertion into the IVN GRA2 associates with GRA6 (85). With the loss of the IVN tubular membranes in the Δ*gra2* background (83), GRA6 may not remain associated with the collapsed IVN membranes present in the Δ*gra2* PV lumen (55). Indeed, recent evidence has shown that GRA6 re-localizes to the PVM in Δ*gra2* parasites (42). Moreover, the C-terminal domain of GRA6 that contains the C-terminal localized HF10 epitope is topologically exposed on exterior face of the PVM after this re-localization occurs in Δ*gra2* PVs (80). Thus, in Δ*gra2* parasites, the GRA6 protein is exposed on the host cytosolic face of the PVM rather than being contained within the PV lumen in association with IVN membranes. These observations are mechanistically important because the GRA6 protein has been reported to be a potent activator of the host cell NFAT4 signaling pathway (86). The re-localization of GRA6 to the PVM could be essential for activation of the host cell NFAT4 signaling pathway, thereby increasing antigen presentation observed in Δ*gra2* parasites. Additional studies are needed to determine the specific role of GRA6 and host NFAT4 signaling in the antigen presentation phenotypes of parasite mutants that lack expression of secreted ROP or GRA proteins.

GRA12 significantly suppressed antigen presentation in infected macrophages and dendritic cells. GRA12's influence on antigen presentation was not strongly dependent on IFN-γ stimulation, IRG effectors, or cell type. GRA12 is another protein that selectively localizes with the IVN (54). The GRA12 molecule was previously identified to associate with ROP5/ROP18 PVM associated high molecular weight protein complexes (25). We recently reported that GRA12 was required for resistance to host IFN-γ, but not for resistance to IRG coating of the PVM (87). Our results show that PVM localized GRA proteins (GRA3, GRA5, and GRA7), and IVN localized GRA proteins (GRA2 and GRA12) suppressed antigen presentation of PV localized parasite antigens by MHC-I. Suggesting that the PV membranes, the PVM and the IVN membranes, actively and dynamically dampen antigen presentation by MHC-I in parasite-infected host cells. In addition, as discussed above for GRA6, if the IVN membrane structure is not established, which is the case in Δ*gra2* parasites (83), antigen presentation by MHC-I is increased through a GRA6-dependent mechanism. Thus, it remains possible that the GRA6 protein underpins a general parasite, or host strategy, to enhance CD8^+^ T cell recognition of parasite-infected cells with ruptured PVs, or PVs that contain altered or damaged PV membranes, the PVM or the IVN membranes. Importantly, the mechanisms that dampen antigen presentation by MHC-I could play an important role in establishing the latent infection, by reducing the CD8^+^ T cell responses that prevent or control the latent infection. Interestingly, the GRA6 protein was recently characterized to also possess a novel N-terminal domain localized MHC-I epitope that elicits CD8^+^ T cells with the capability to eliminate latent cysts in the brain (88). All together, these findings suggest that active suppression of MHC-I antigen presentation by secreted ROP and GRA proteins is likely to be important for the parasites ability to establish and maintain the latent infection.

*Toxoplasma* infection also actively suppresses MHC-II presentation by antigen presenting cells (89–92). However, multiple reports indicate that *Toxoplasma* infected antigen presenting cells are not severely compromised and could even be activated in their ability to express MHC-I and associated co-stimulatory molecules (CD80, CD86, or CD40) to functionally present antigen by MHC-I to activate CD8^+^ T cells (3–5, 13, 28, 30, 89, 93, 94). *Toxoplasma* secreted effectors such as ROP5, ROP18, and GRA7 resist cell autonomous killing mechanisms to maintain vacuole integrity and parasite survival while also reducing or delaying host access to PV antigens for CD8^+^ T cell recognition. Furthermore, our study highlights that ROP5, ROP18, GRA2, GRA3, GRA7, and GRA12 also function to dampen, but not to completely ablate, presentation of parasite antigens by MHC-I. Moreover, the IRG independent as well as IRG dependent GRA2 antigen presentation phenotypes were co-dependent on the expression of the GRA6 molecule. Thus, it remains to be determined whether all of the antigen presentation phenotypes associated with ROP5, ROP18, GRA2, GRA3, GRA7, and GRA12 molecules identified in our study arise from the re-localization or release of GRA6 and subsequent activation of the host cell NFAT4 signaling pathway(s) that leads to increased presentation of PV antigens by MHC-I.

Collectively, our findings argue for potent recognition of intracellular *Toxoplasma* by antigen presenting cells to activate strong CD8^+^ T cell responses, and indeed, potent CD8^+^ responses are stimulated following vaccination with live-attenuated non-replicating uracil auxotroph mutants of *Toxoplasma* (5, 7, 27, 28, 95–101). Consequently, if *Toxoplasma* failed to modulate the host CD8^+^ T cell responses, the parasite infection could be cleared before latent infection was established, and if the parasite dampened the CD8^+^ T cell responses too effectively then the host could succumb from parasite replication and tissue damage or associated inflammation, and consequently, latent infection would not be established (102). To mediate this dynamic host manipulation, the IVN membrane system in the PV lumen could represent a sensor to detect PV membrane perturbations. PV membrane perturbations could trigger re-localization of GRA6 to the PVM in still intact vacuoles, or release GRA6 into the host cytosol. Our findings suggest that the *Toxoplasma* GRA6 protein underpins a key mechanism that enhances CD8^+^ T cell recognition of parasite-infected cells with ruptured PVs, or PVs with damaged PV membranes. In contrast, intact PVs actively and effectively resist the host cells attempt to access and present PV localized antigens by MHC-I. This parasite modulation of host access to PV antigens and their presentation by MHC-I is likely to represent an adaptive mechanism to fine-tune the host CD8^+^ T cell responses to promote host survival as well as to establish successful latent infection, thereby increasing parasite transmission. In sum, our data shows that rhoptry and dense granule secreted proteins that associate with the parasitophorous vacuole membrane or the intravacuolar network membranes play important roles to dynamically regulate CD8^+^ T cell recognition of *Toxoplasma* infected host cells.

## Data Availability

All datasets generated for this study are included in the manuscript/Supplementary Files.

## Ethics Statement

The animal study was reviewed and approved by Institutional Animal Care and Use Committee of Dartmouth College (Animal Welfare Assurance Number #A3259-01).

## Author Contributions

LR, BF, and DB: conceived and designed the experiments. LR, BF, KB, VC, and DB: performed the experiments. LR, BF, KB, GT, and DB: analyzed the data. LR, BF, GT, and DB: wrote the paper.

### Conflict of Interest Statement

The authors declare that the research was conducted in the absence of any commercial or financial relationships that could be construed as a potential conflict of interest.

## Supplementary Material

Figure S1 Development of OVA-secreting *Toxoplasma* knock out strains. **(A)** Strategy to generate isogenic OVA expressing *Toxoplasma* by targeting insertion of OVA into the *UPRT* locus via 5-fluorodeoxyuridine (FUDR) selection and double homologous recombination. **(B)** Validation of OVA insertion by PCR. Six of six clones (1-6) were positive for both PCR1 (~1,300 bp) and PCR2 (~1,600 bp) (see Figure 1A) showing correct 5′ and 3′ integration of OVA into the *UPRT* locus. M = marker. **(C)** Immunofluorescence validation of OVA expression (green) by *Toxoplasma* in HFF cells infected for 48 h *in vitro*. **(D)** Validation of OVA expression (green) by immunofluorescence imaging of OVA expressing strains that lack (Δ) a ROP or GRA gene as indicated. Complemented strains are indicated (::).

Figure S2 Complementation and validation of the Δ*rop5 and* Δ*gra2* knockout strains. **(A)** The strategy for complementation of Δ*rop5* at it's endogenous with the WT *ROP5C* allele using 6-thioxanthine selection. **(B)** PCR validation of Δ*rop5::ROP5C. Six* of six clones were positive for both PCR1 (~3,100 bp) and PCR2 (~3,000 bp). **(C)** Immunofluorescence validation of Δ*rop5::ROP5C*. HFF cells were infected for 24 h then fixed with 4% PFA, permeabilized with Triton X-100 and blocked with 10% FBS, cells were stained with anti-rop5 (Kindly donated by the Sibley Lab) (secondary: Alexa Fluor 488). **(D)** The strategy for complementation of Δ*gra2* at it's endogenous with the WT *GRA2* allele using 6TX selection. **(E)** PCR validation of Δ*gra2::GRA2. Six* of six clones were positive for both PCR1 (~2,100 bp) and PCR2 (~1,400 bp). **(F)** Immunofluorescence validation of Δ*gra2::GRA2*. HFF cells were infected with parasites for 24 h then fixed with 4% PFA, permeabilized with Triton X-100 and blocked with 10% FBS, cells were stained with anti-gra2 (Kindly donated by the Cesbron-Delauw lab) (secondary: Alexa Fluor 488).

Figure S3 Complementation and validation of Δ*rop18* knockout strain. **(A)** Strategy for complementation of Δ*rop18* at the *UPRT* locus with the WT allele (*ROP18*), a kinase dead mutant (*ROP18*^*KD*^), and an ATF6β binding motif mutant (*ROP18*^*ATF*6^^β^) using 5-fluordeoxyuridine selection. **(B)** PCR validation of Δ*rop18::ROP18*. Six of six clones were positive for both PCR1 (~2,100 bp) and PCR2 (~1,600 bp). **(C)** PCR validation of Δ*rop18::ROP18*^*KD*^. Six of six clones were positive for both PCR1 (~2,100 bp) and PCR2 (~1,600 bp). **(D)** PCR validation of Δ*rop18::ROP18*^*ATF*6^^β^. Six of six clones were positive for both PCR1 (~2,050 bp) and PCR2 (~1,600 bp). **(E)** Immunofluorescence validation of Δ*rop18::ROP18*, Δ*rop18::ROP18*^*KD*^, and Δ*rop18::ROP18*^*ATF*6^^β^. HFF cells were infected for 24 h and ROP18 was visualized with anti-rop18 (secondary: Alexa Fluor 488).

Figure S4 *Toxoplasma* infected macrophages and dendritic cells express equivalent OVA antigen in the PV lumen. **(A)** To verify equivalent expression of OVA in the PV lumen, BMMΦs, and **(B)** BMDCs were infected with different *Toxoplasma* isogneic strains at an MOI of 2.5 and incubated for 22–24 h prior to fixation with PFA. Cells were then permeabilized with 0.05% saponin and stained for anti-GRA5 and anti-OVA. Samples were analyzed by FACS and gated on the cells that were double positive for GRA5 and OVA.

Figure S5 The *ROP5C* allele partially complements the virulence defect in Δ*rop5*. Survival data for female C57BL/6 mice infected with either 100,000 or 1,000 tachyzoites of the shown strains (*n* = 4). Gehan-Breslow-Wilcoxon Test. ***P* < 0.005, **P* < 0.05, ns = not significant.

Figure S6 Heat map summary of the effect of ROP or GRA deletion on antigen presentation by MHC-I. Antigen presentation data from the B3Z assay is represented as graphical summary plot. Color indicates the fold change increase in antigen presentation over unprimed wildtype antigen presenting cells infected with the *Toxoplasma* RH strain not expressing OVA.

Table S1 *Toxoplasma gondii* strains used or developed in this study.

Table S2 Primers for generating targeted insertions or deletions.

Table S3 Validation primers.
