# Supplementary figures and images for: Rhoptry and Dense Granule Secreted Effectors Regulate CD8+ T Cell Recognition of Toxoplasma gondii Infected Host Cells
Source: Front Immunol. 2019 Sep 6;10:2104. doi: 10.3389/fimmu.2019.02104 (PMC6742963; doi:10.3389/fimmu.2019.02104)

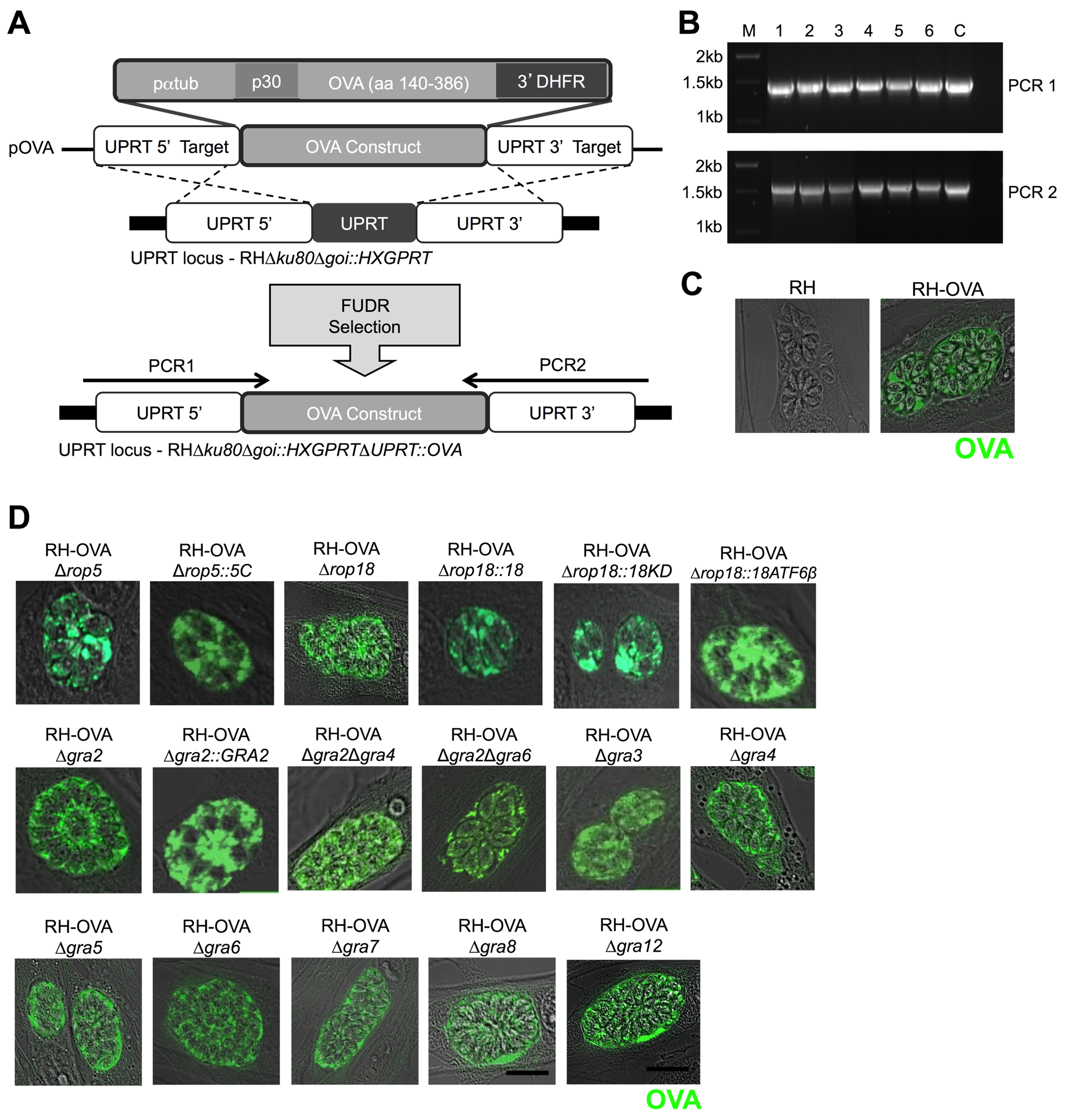

Supplement: Figure S1 — Development of OVA-secreting Toxoplasma knock out strains. (A) Strategy to generate isogenic OVA expressing Toxoplasma by targeting insertion of OVA into the UPRT locus via 5-fluorodeoxyuridine (FUDR) selection and double homologous recombination. (B) Validation of OVA insertion by PCR. Six of six clones (1-6) were positive for both PCR1 (~1,300 bp) and PCR2 (~1,600 bp) (see Figure 1A) showing correct 5′ and 3′ integration of OVA into the UPRT locus. M = marker. (C) Immunofluorescence validation of OVA expression (green) by Toxoplasma in HFF cells infected for 48 h in vitro. (D) Validation of OVA expression (green) by immunofluorescence imaging of OVA expressing strains that lack (Δ) a ROP or GRA gene as indicated. Complemented strains are indicated (::). [file Image_1.TIF]

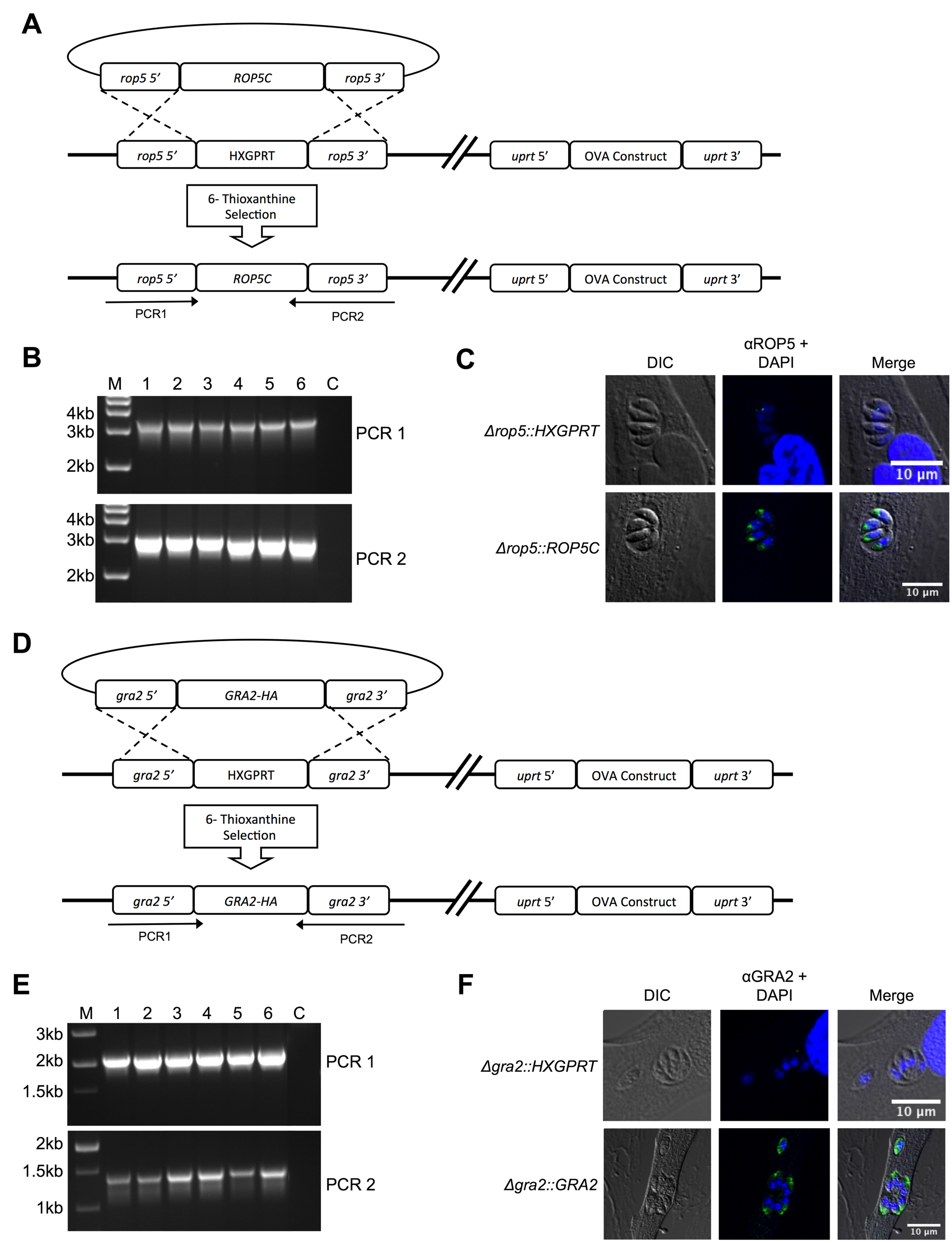

Supplement: Figure S2 — Complementation and validation of the Δrop5 and Δgra2 knockout strains. (A) The strategy for complementation of Δrop5 at it's endogenous with the WT ROP5C allele using 6-thioxanthine selection. (B) PCR validation of Δrop5::ROP5C. Six of six clones were positive for both PCR1 (~3,100 bp) and PCR2 (~3,000 bp). (C) Immunofluorescence validation of Δrop5::ROP5C. HFF cells were infected for 24 h then fixed with 4% PFA, permeabilized with Triton X-100 and blocked with 10% FBS, cells were stained with anti-rop5 (Kindly donated by the Sibley Lab) (secondary: Alexa Fluor 488). (D) The strategy for complementation of Δgra2 at it's endogenous with the WT GRA2 allele using 6TX selection. (E) PCR validation of Δgra2::GRA2. Six of six clones were positive for both PCR1 (~2,100 bp) and PCR2 (~1,400 bp). (F) Immunofluorescence validation of Δgra2::GRA2. HFF cells were infected with parasites for 24 h then fixed with 4% PFA, permeabilized with Triton X-100 and blocked with 10% FBS, cells were stained with anti-gra2 (Kindly donated by the Cesbron-Delauw lab) (secondary: Alexa Fluor 488). [file Image_2.TIF]

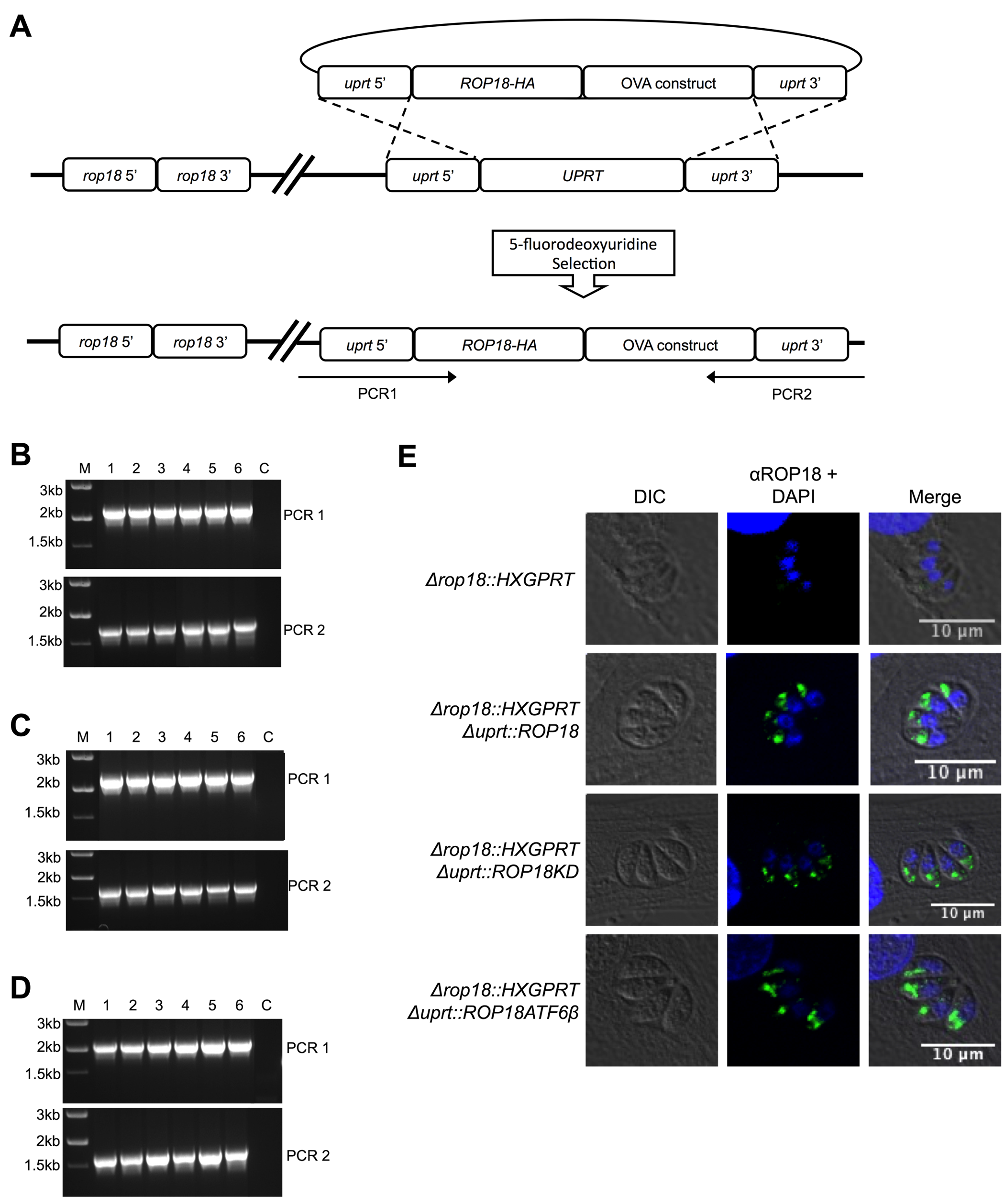

Supplement: Figure S3 — Complementation and validation of Δrop18 knockout strain. (A) Strategy for complementation of Δrop18 at the UPRT locus with the WT allele (ROP18), a kinase dead mutant (ROP18KD), and an ATF6β binding motif mutant (ROP18ATF6β) using 5-fluordeoxyuridine selection. (B) PCR validation of Δrop18::ROP18. Six of six clones were positive for both PCR1 (~2,100 bp) and PCR2 (~1,600 bp). (C) PCR validation of Δrop18::ROP18KD. Six of six clones were positive for both PCR1 (~2,100 bp) and PCR2 (~1,600 bp). (D) PCR validation of Δrop18::ROP18ATF6β. Six of six clones were positive for both PCR1 (~2,050 bp) and PCR2 (~1,600 bp). (E) Immunofluorescence validation of Δrop18::ROP18, Δrop18::ROP18KD, and Δrop18::ROP18ATF6β. HFF cells were infected for 24 h and ROP18 was visualized with anti-rop18 (secondary: Alexa Fluor 488). [file Image_3.TIF]

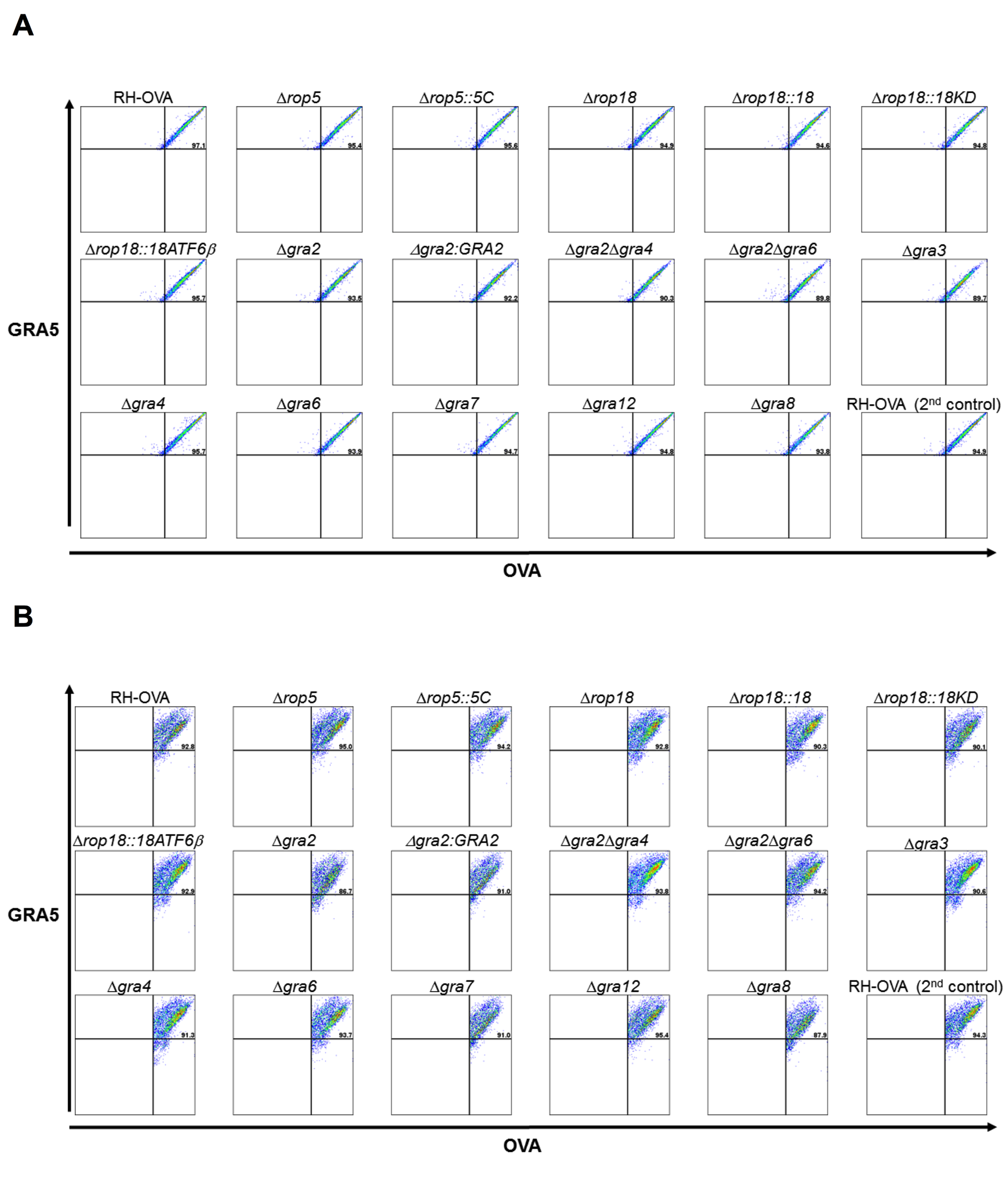

Supplement: Figure S4 — Toxoplasma infected macrophages and dendritic cells express equivalent OVA antigen in the PV lumen. (A) To verify equivalent expression of OVA in the PV lumen, BMMΦs, and (B) BMDCs were infected with different Toxoplasma isogneic strains at an MOI of 2.5 and incubated for 22–24 h prior to fixation with PFA. Cells were then permeabilized with 0.05% saponin and stained for anti-GRA5 and anti-OVA. Samples were analyzed by FACS and gated on the cells that were double positive for GRA5 and OVA. [file Image_4.TIF]

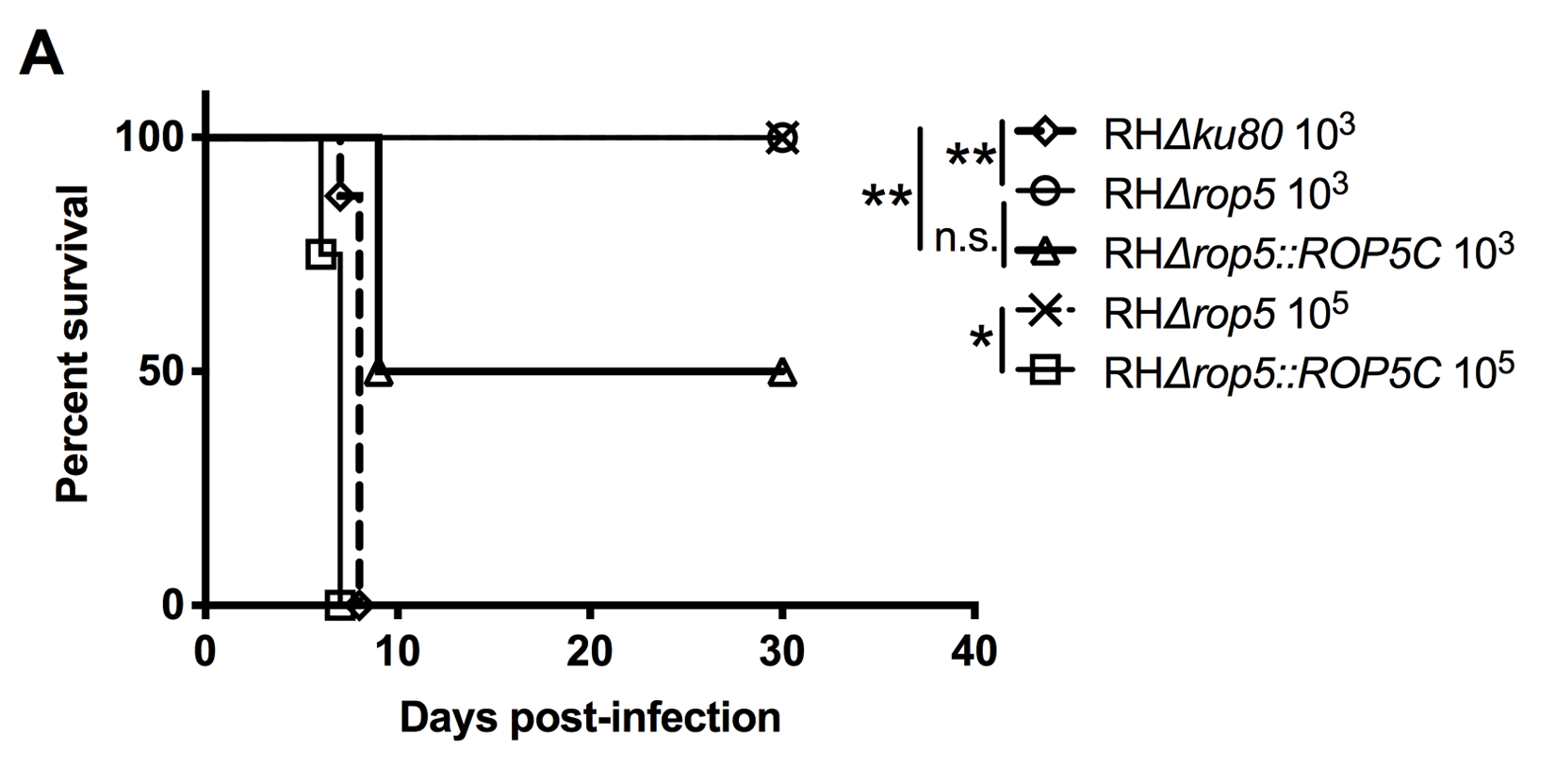

Supplement: Figure S5 — The ROP5C allele partially complements the virulence defect in Δrop5. Survival data for female C57BL/6 mice infected with either 100,000 or 1,000 tachyzoites of the shown strains (n = 4). Gehan-Breslow-Wilcoxon Test. **P < 0.005, *P < 0.05, ns = not significant. [file Image_5.TIF]

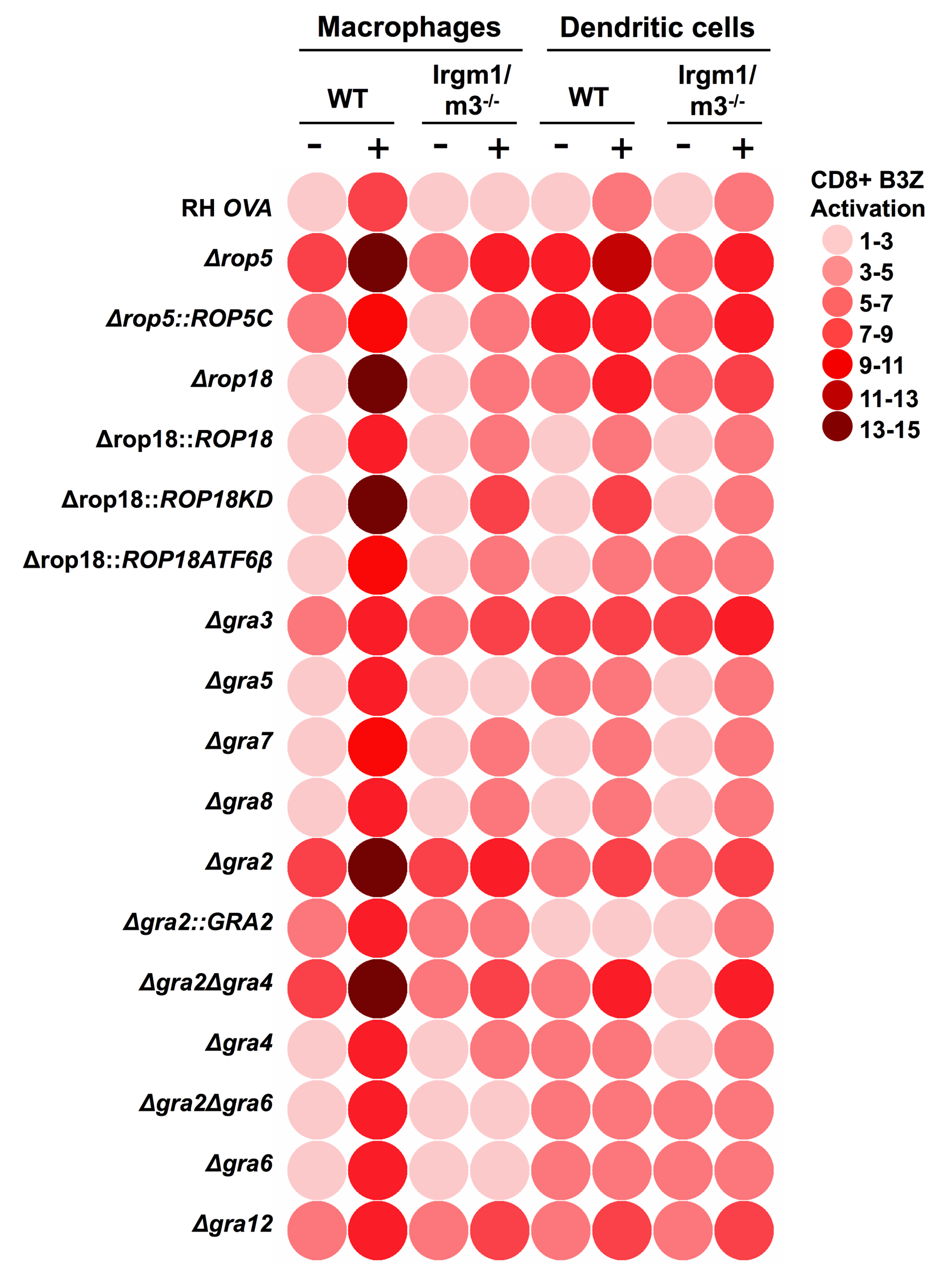

Supplement: Figure S6 — Heat map summary of the effect of ROP or GRA deletion on antigen presentation by MHC-I. Antigen presentation data from the B3Z assay is represented as graphical summary plot. Color indicates the fold change increase in antigen presentation over unprimed wildtype antigen presenting cells infected with the Toxoplasma RH strain not expressing OVA. [file Image_6.TIF]
