# Supplementary material for: Rhoptry and Dense Granule Secreted Effectors Regulate CD8+ T Cell Recognition of Toxoplasma gondii Infected Host Cells
Source: Front Immunol. 2019 Sep 6;10:2104. doi: 10.3389/fimmu.2019.02104 (PMC6742963; doi:10.3389/fimmu.2019.02104)
Supplement: Table S1 — Toxoplasma gondii strains used or developed in this study. [file Data_Sheet_1.docx]

**Table S1. *Toxoplasma gondii* strains used or developed in this study.**

| **Strain** | **Parent Strain** | **Genotype** |
| --- | --- | --- |
| RH*Δku80::HXGPRT^a^* |  | *Δku80* |
| RH*Δku80Δhxgprt^a^* | RH*Δku80::HXGPRT* | *Δku80Δhxgprt* |
| RH*Δku80ΔhxgprtΔuprt::OVA* | RH*Δku80Δhxgprt^a^* | *Δku80ΔhxgprtΔuprt::OVA* |
| RH*Δku80Δrop5::HXGPRTΔuprt::OVA* | RH*Δku80Δrop5::HXGPRT* | *Δku80Δrop5::HXGPRTΔuprt::OVA* |
| RH*Δku80Δrop5::ROP5CΔuprt::OVA* | RH*Δku80Δrop5:: HXGPRTΔuprt::OVA* | *Δku80Δrop5::ROP5CΔuprt::OVA* |
| RH*Δku80Δrop18::HXGPRTΔuprt::OVA* | RH*Δku80Δrop18::HXGPRT^b^* | *Δku80Δrop18::HXGPRTΔuprt::OVA* |
| RH*Δku80Δrop18::HXGPRTΔuprt::ROP18OVA* | RH*Δku80Δrop18::HXGPRT* | *Δku80Δrop18::HXGPRTΔuprt::ROP18OVA* |
| RH*Δku80Δrop18::HXGPRTΔuprt::ROP18^KD^OVA* | RH*Δku80Δrop18::HXGPRT* | *Δku80Δrop18::HXGPRTΔuprt::ROP18^KD^OVA* |
| RH*Δku80Δrop18::HXGPRTΔuprt:: ROP18^ATF6β^OVA* | RH*Δku80Δrop18::HXGPRT* | *Δku80Δrop18::HXGPRTΔuprt::ROP18^ATF6β^OVA* |
| RH*Δku80Δgra2::HXGPRTΔuprt::OVA* | RH*Δku80Δgra2::HXGPRT^c^* | *Δku80Δgra2::HXGPRTΔuprt::OVA* |
| RH*Δku80Δgra2::GRA2Δuprt::OVA* | RH*Δku80Δgra2::HXGPRTΔuprt::OVA* | *Δku80Δgra2::GRA2Δuprt::OVA* |
| RH*Δku80Δgra3::HXGPRTΔuprt::OVA* | RH*Δku80Δgra3::HXGPRT^c^* | *Δku80Δgra3::HXGPRTΔuprt::OVA* |
| RH*Δku80Δgra4::HXGPRTΔuprt::OVA* | RH*Δku80Δgra4::HXGPRT^c^* | *Δku80Δgra4::HXGPRTΔuprt::OVA* |
| RH*Δku80Δgra5::HXGPRTΔuprt::OVA* | RH*Δku80Δgra5::HXGPRT^c^* | *Δku80Δgra5::HXGPRTΔuprt::OVA* |
| RH*Δku80Δgra6::HXGPRTΔuprt::OVA* | RH*Δku80Δgra6::HXGPRT^c^* | *Δku80Δgra6::HXGPRTΔuprt::OVA* |
| RH*Δku80Δgra7::HXGPRTΔuprt::OVA* | RH*Δku80Δgra7::HXGPRT^c^* | *Δku80Δgra7::HXGPRTΔuprt::OVA* |
| RH*Δku80Δgra8::HXGPRTΔuprt::OVA* | RH*Δku80Δgra8::HXGPRT^c^* | *Δku80Δgra8::HXGPRTΔuprt::OVA* |
| RH*Δku80Δgra12::HXGPRT* | RH*Δku80Δhxgprt^a^* | *Δku80Δgra12::HXGPRT* |
| RH*Δku80Δgra12::HXGPRTΔuprt::OVA* | RH*Δku80Δgra12::HXGPRT* | *Δku80Δgra12::HXGPRTΔuprt::OVA* |
| RH*Δku80Δgra2Δgra4::HXGPRTΔuprt::OVA* | RH*Δku80Δgra2Δgra4::HXGPRT^c^* | *Δku80 Δgra2Δgra4::HXGPRTΔuprt::OVA* |
| RH*Δku80Δgra2Δgra6::HXGPRTΔuprt::OVA* | RH*Δku80Δgra2Δgra6::HXGPRT^c^* | *Δku80 Δgra2Δgra6::HXGPRTΔuprt::OVA* |

*^a^* Fox, B.A., *et al.,* 2009 *Eukaryotic Cell*

*^b^* Fentress, S., *et al.,* 2010 *Cell Host Microbe*

*^c^* Rommereim, L.M., *et al.,* 2016 *PLoS One*

**Table S2. Primers for generating targeted insertions or deletions.**

| **PCR Product** | 5’ target | 3’ target | OVA | OVA | 5’ target | 3’ target | 5' target | 5' target | 3' target | 3' target | 5’ target | 3’ target | Coding Region | Coding Region | 5’ target | 3’ target | Coding region | Coding region | ATF6β Deletion | ATF6β Deletion |
| --- | --- | --- | --- | --- | --- | --- | --- | --- | --- | --- | --- | --- | --- | --- | --- | --- | --- | --- | --- | --- |
| **Use** | Construction of pOVA | Construction of pOVA | Construction of pOVA | Construction of pOVA | Construction of UPRT plasmids | Construction of UPRT plasmids | Construction of pΔROP5 | Construction of pΔROP5 | Construction of pΔROP5 | Construction of pΔROP5 | Construction of type I pΔROP5::ROP5C | Construction of type I pΔROP5::ROP5C | Construction of type I pΔROP5::ROP5C | Construction of type I pΔROP5::ROP5C | Construction of pΔROP18::ROP18OVA | Construction of pΔROP18::ROP18OVA | Construction of pΔROP18::ROP18OVA | Construction of pΔROP18::ROP18OVA | Construction of pΔROP18::ROP18ATFOVA | Construction of pΔROP18::ROP18ATFOVA |
| **Sequence** | GCTCATAACCGTACCACGTGAAATAGTAGCTCTGACTAGTAGCTTTCCGCTCGCTGGGAC | GTTGACAAGTGTTCTGGCAGGCTACAGTGACAACTAGTGCCCTGCTGTCTTGTCAGGTACT | TGGCGCAGGTCCCAGCGAGCGGAAAGCTACTAGTCAGAGCTACTATTTCACGTGGTACGG | ATGGTTCCAAAGTACCTGACAAGACAGCAGGGCACTAGTGGTGTCACTGTAGCCTGCCAG | TTGGGTAACGCCAGGGTTTTCCCAGTCACGACGCTCGAGGTGAGCTCATGCTGGAGCTTCG | GTGAGCGGATAACAATTTCACACAGGAAACAGCGCGGCCGCCTGGCGTTCGATCGACCGAAG | GCGGGTTTGAATGCAAGGTTTCGTGCTGATCAAACTAGTGGCATTGCAGAATCTATGCAGCCAG | TTGGGTAACGCCAGGGTTTTCCCAGTCACGACGGTTTAAACGAGAAGATACTGATGTGCTGCACACG | TTCTGGCAGGCTACAGTGACACCGCGGTGGAGGACTAGTTCCACTCACTGGTGTAGTCGATGC | GTGAGCGGATAACAATTTCACACAGGAAACAGCGCGGCCGCGTGTAGCGTGCCACACTTCGC | CATACTACAACCTGGCTGCATAGATTCTGCAATGCCACTAGTAGCTGTTAGAGTCGCGACCATGG | CGTCTTCGTGCATCGACTACACCAGTGAGTGGAACTAGTCAGATACTGTACACACGACTACCAGC | AGCTGTTAGAGTCGCGACCATGG | CAGATACTGTACACACGACTACCAGC | CAAGAGGTGCATAGCGTGGCTAGCGTTTAAACAGCTTTCCGCTCGCTGGGACCTGCGCCATTTTAG | CCATGTTGTTCGTATCGACTCTGCGGTTTAAACCAGAGCTACTATTTCACGTGGTACGGTTATGAG | CTAAAATGGCGCAGGTCCCAGCGAGCGGAAAGCTGTTTAAACGCTAGCCACGCTATGCACCTCTTG | TACCCATACGATGTTCCAGATTACGCTTAAAAGACTCAAATGAAAAGGGGAACGTGGCGG | GCAGCTGATAGCTCCTCGAGACCCCGTCTGTCCGGAATCCGGAGGTACTTTCCTCAAGGGC | GCCCTTGAGGAAAGTACCTCCGGATTCCGGACAGACGGGGTCTCGAGGAGCTATCAGCTGC |
| **Primer Name** | ptub_R | DHFR.3’_F | secOVA_F | secOVA_R | UPRTF1 | UPRTR2 | ROP5R1 | ROP5F1 | ROP5F2 | ROP5R2 | ROP5_ROP5C_R1 | ROP5_ROP5C_F2 | ROP5C_F | ROP5C_R | UPRT_ROP18_R1 | ROP18_OVA_F | ROP18_CR_F | ROP18_CR.HA_R | ROP18_ATF_F | ROP18_ATF_R |

**Table S2. Primers for generating targeted insertions or deletions (cont.)**

| **PCR Product** | 5’ target | 5’ target | 3’ target | 3’ target | Coding Region | Coding Region | 5' target | 5' target | 3' target | 3' target |
| --- | --- | --- | --- | --- | --- | --- | --- | --- | --- | --- |
| **Use** | Construction of pΔGRA2::GRA2 | Construction of pΔGRA2::GRA2 | Construction of pΔGRA2::GRA2 | Construction of pΔGRA2::GRA2 | Construction of pΔGRA2::GRA2 | Construction of pΔGRA2::GRA2 | Construction of pΔGRA12 | Construction of pΔGRA12 | Construction of pΔGRA12 | Construction of pΔGRA12 |
| **Sequence** | TTGGGTAACGCCAGGGTTTTCCCAGTCACGACGGTTTAAACCTATGGAACAAGCCGTGTGTCGATAG | GCGGGTTTGAATGCAAGGTTTCGTGCTGATCAAACTAGTTCGCACTTAGCCTAAGGGACGAC | TAATGTTGACTACGACGAAAGTGATGCGC | GTGAGCGGATAACAATTTCACACAGGAAACAGCGCGGCCGCCGCCCTCGTGATTCCTCCAAG | GCCTGCGCATCACTTTCGTCGTAGTCAACATTAAGCGTAATCTGGAACGTCATATGGATAGGATCCTGCATAGTCCGGGACGTCATAGGGATAGCCCGCATAGTCAGGAACATCGTATGGGTACTGCGAAAAGTCTGGGACGGG | GTCGTCCCTTAGGCTAAGTGCGA | TTGGGTAACGCCAGGGTTTTCCCAGTCACGACGGTTTAAACCCGACGACATCTTGGTCACACC | GCGGGTTTGAATGCAAGGTTTCGTGCTGATCAAACTAGTACTGGCAGGCACTCGATAGGG | TTCTGGCAGGCTACAGTGACACCGCGGTGGAGGACTAGTGTTGGAGCAGCTCTTGCTCGAG | GTGAGCGGATAACAATTTCACACAGGAAACAGCGCGGCCGCCCATCTCCCATTGTTGAAAGAGTGCG |
| **Primer Name** | GRA2F1 | GRA2R1 | GRA2F2 | GRA2R2 | GRA2CR.HA.R | GRA2CR_F | GRA12F1 | GRA12R1 | GRA12F2 | GRA12R2 |

**Table S3. Validation primers.**
